# Supplementary material for: aPKC Phosphorylates p27Xic1, Providing a Mechanistic Link between Apicobasal Polarity and Cell-Cycle Control
Source: Dev Cell. 2014 Dec 8;31(5):559–71. doi: 10.1016/j.devcel.2014.10.023 (PMC4262734; doi:10.1016/j.devcel.2014.10.023)
Supplement: Document S1. Supplemental Experimental Procedures and Figures S1–S6 [file mmc1.pdf]

Developmental Cell, Volume *31*

Supplemental Information

# aPKC Phosphorylates p27Xic1, Providing a Mechanistic Link between Apicobasal Polarity and Cell-Cycle Control

Nitin Sabherwal, Raphael Thuret, Robert Lea, Peter Stanley, and Nancy Papalopulu

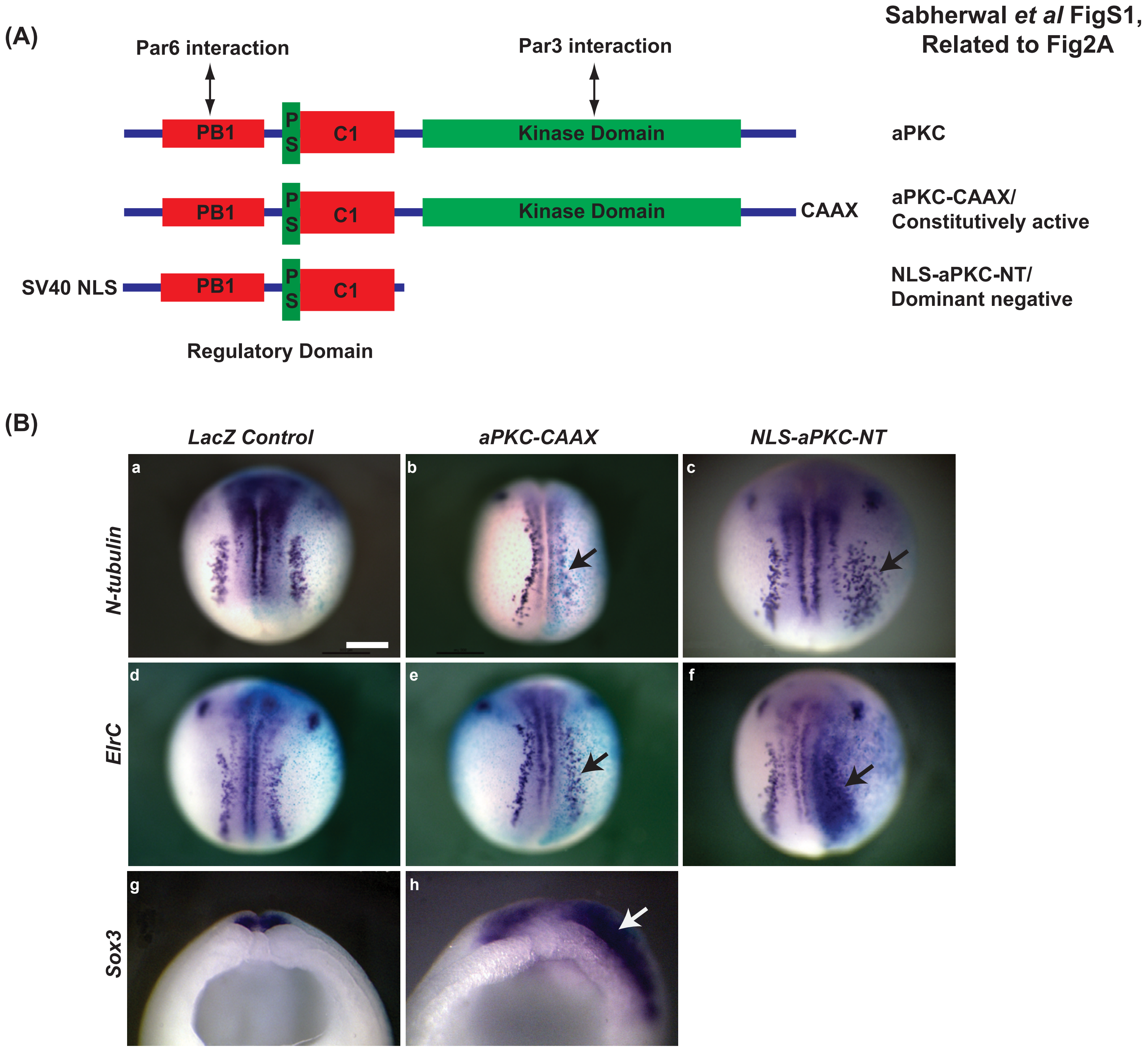


**FigS1, related to fig2A:** Over-expression of apicobasal polarity kinase aPKC promotes progenitor proliferation and suppresses neuronal differentiation. **(A)** shows wild type , constitutively active and dominant negative versions of aPKC with their important domains. PB1 domain (responsible for aPKC's interaction with Par6), PS/pseudosubstrate domain and C1 domain (responsible for its interaction with membrane lipids) constitute aPKC's N-terminal regulatory domain, while C terminal part of the protein constitutes its kinase domain. The schematic has been adapted from Suzuki et al, 2003, J. Biochem. 133, 9–16. Constitutively active and dominant negative forms of aPKC have been reported in our previous publication (Sabherwal *et al*, 2009). Overexpression of membrane targeted active aPKC (*aPKC-CAAX*) suppresses neuronal differentiation as differentiation markers *N-tubulin* and *ElrC* showed reduction on injected side of the embryos **(Bb and Be)**, and promoted neural progenitor proliferation (Progenitor marker *Sox3* was enhanced on injected side, **Bh**). Overexpressing nuclear dominant negative form of aPKC had the inverse effect of promoting differentiation **(Bc and Bf)**. Scale bar in Ba represents 500μM.

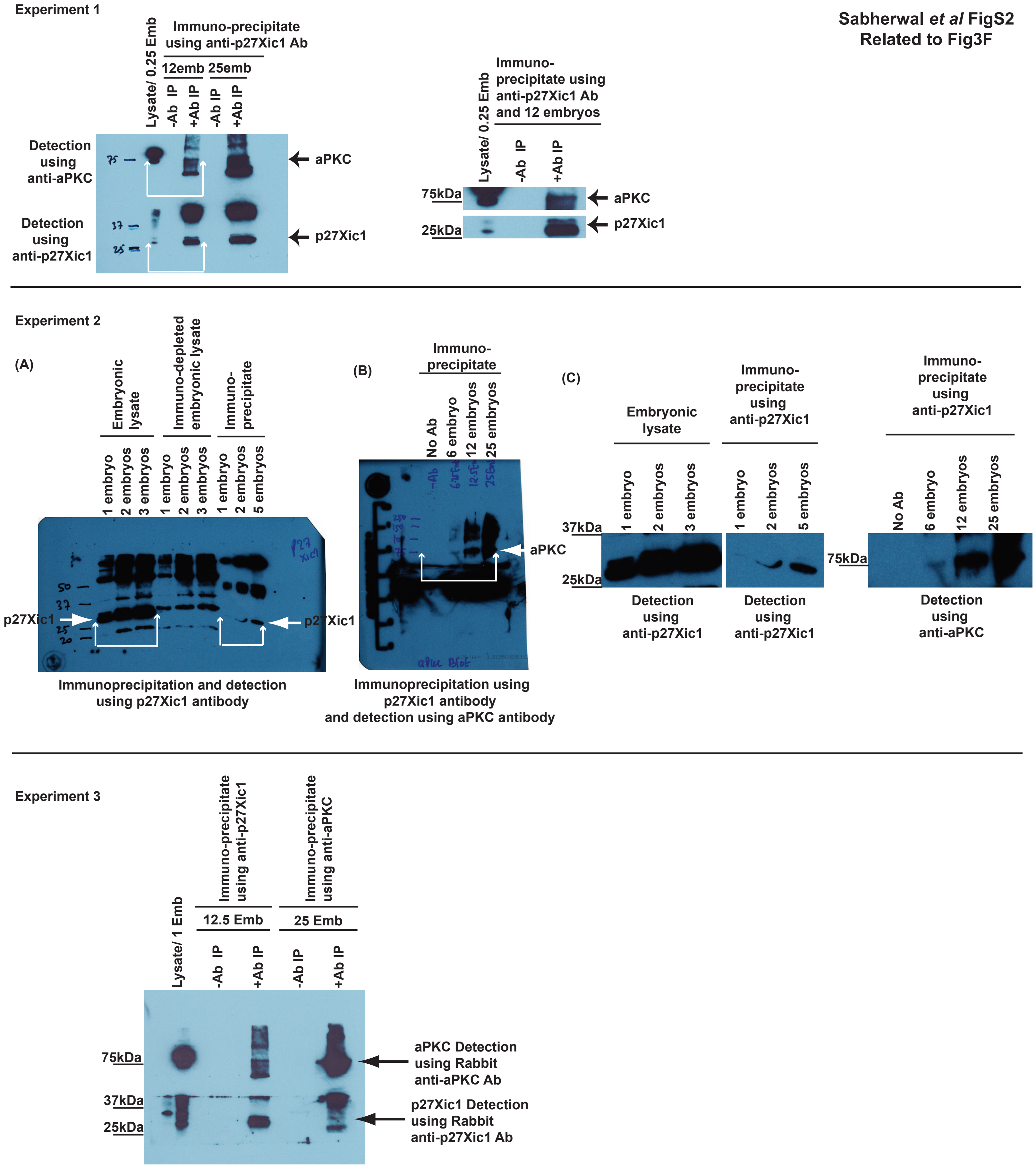


**FigS2, related to fig3F:** Physical interaction between p27Xic1 and aPKC was confirmed for endogenous proteins from embryonic lysates by a co-immuno-precipitation assay using antibodies against endogenous p27Xic1 and aPKC. In the first two experiments and last experiment (lanes 2 and 3), p27Xic1 was immuno-precipitated from embryonic lysate usinga home-made antibody against p27Xic1 and aPKC bound to p27Xic1 was detected using a commercially available aPKC antibody. Original blots for first two experiments are shown on the left hand side and the final blots are shown on right hand side. Final blot from the experiment 1 has been shown in fig3F. The last two lanes in experiment 3 show the inverse experiment where aPKC was immunoprecipitated using antibody against aPKC and p27Xic1 bound to it was detected using home-made antibody against p27Xic1. .

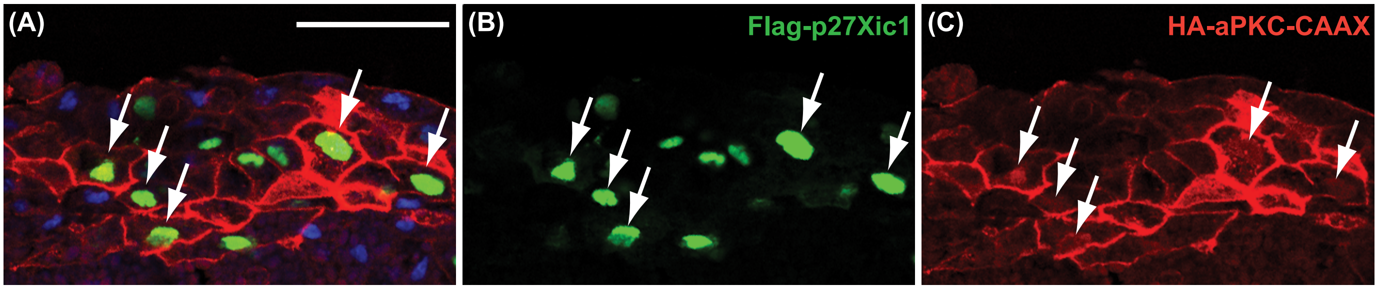


**FigS3, related to fig3:** Regulation of p27Xic1 activity by aPKC likely takes place in the cell nucleus. To see where in the cell p27Xic1 and aPKC-CAAX interact with each other, we overexpressed Flag-p27Xic1 and HA-aPKC-CAAX in *Xenopus* embryos and let them grow until neurula stage. The embryos were fixed, sectioned and immunostainied using anti-Flag and anti-HA antibody for detection of Flag-p27Xic1 and HA-aPKC-CAAX repectively. Immuno-staining showed that Flag-p27Xic1 (green) was largely in the nucleus of the cells, but a portion of aPKC-CAAX (red), which was mostly localized to the cell cortex, was also found in the nucleus (see the white arrows). These stainings led to the idea that aPKC interacts with p27Xic1 in the nucleus. White scale bar in A represents 50μM.

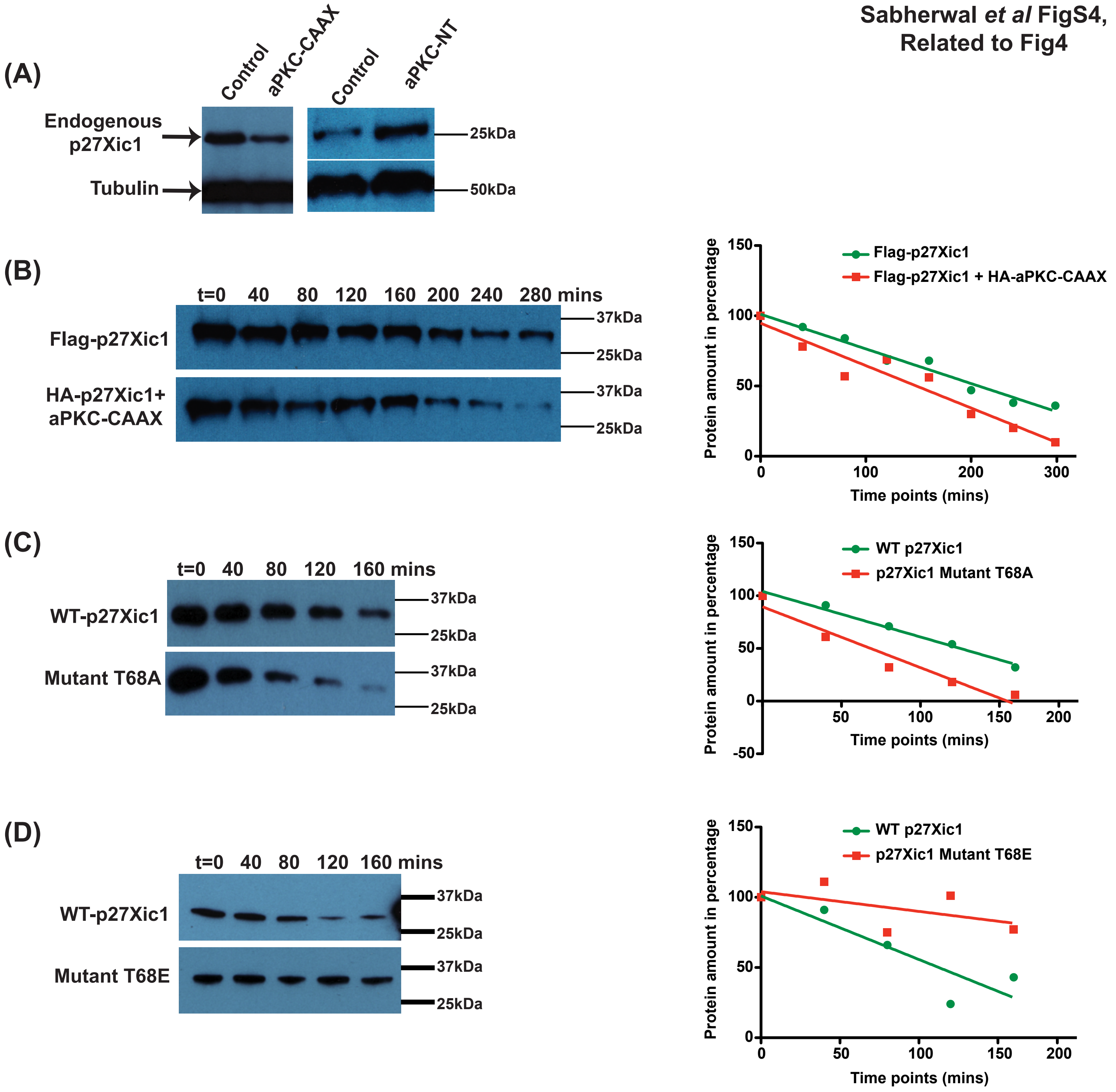


**FigS4, related to fig4:** Effects of aPKC on protein abundance and stability of p27Xic1. **(A)** Embryos over-expressing constitutively active aPKC (aPKC-CAAX) or dominant negative form of aPKC (aPKC-NT) show reduced or increased levels of endogenous p27Xic1, respectively. **(B)** Cycloheximide treatment of HeLa cells overexpressing Flag-p27Xic1 alone or in combination with HA-aPKC-CAAX shows that half-life of p27Xic1 is reduced in the presence of aPKC-CAAX, suggesting that p27Xic1 phosphorylation by aPKC might affect its half-life. **(C)** But contrary to the expectations, phospho-mutant of p27Xic1 (eg. T68A) showed protein destabilization, while protein for its phospho-mimetic mutant (T68E) showed enhanced stability **(D)**, leading to the idea that the effects of aPKC on the stability of p27Xic1 are complex and can not be mimicked by singlepoint mutations on p27Xic1.

Sabherwal *et al* FigS5
Related to Fig4C and D

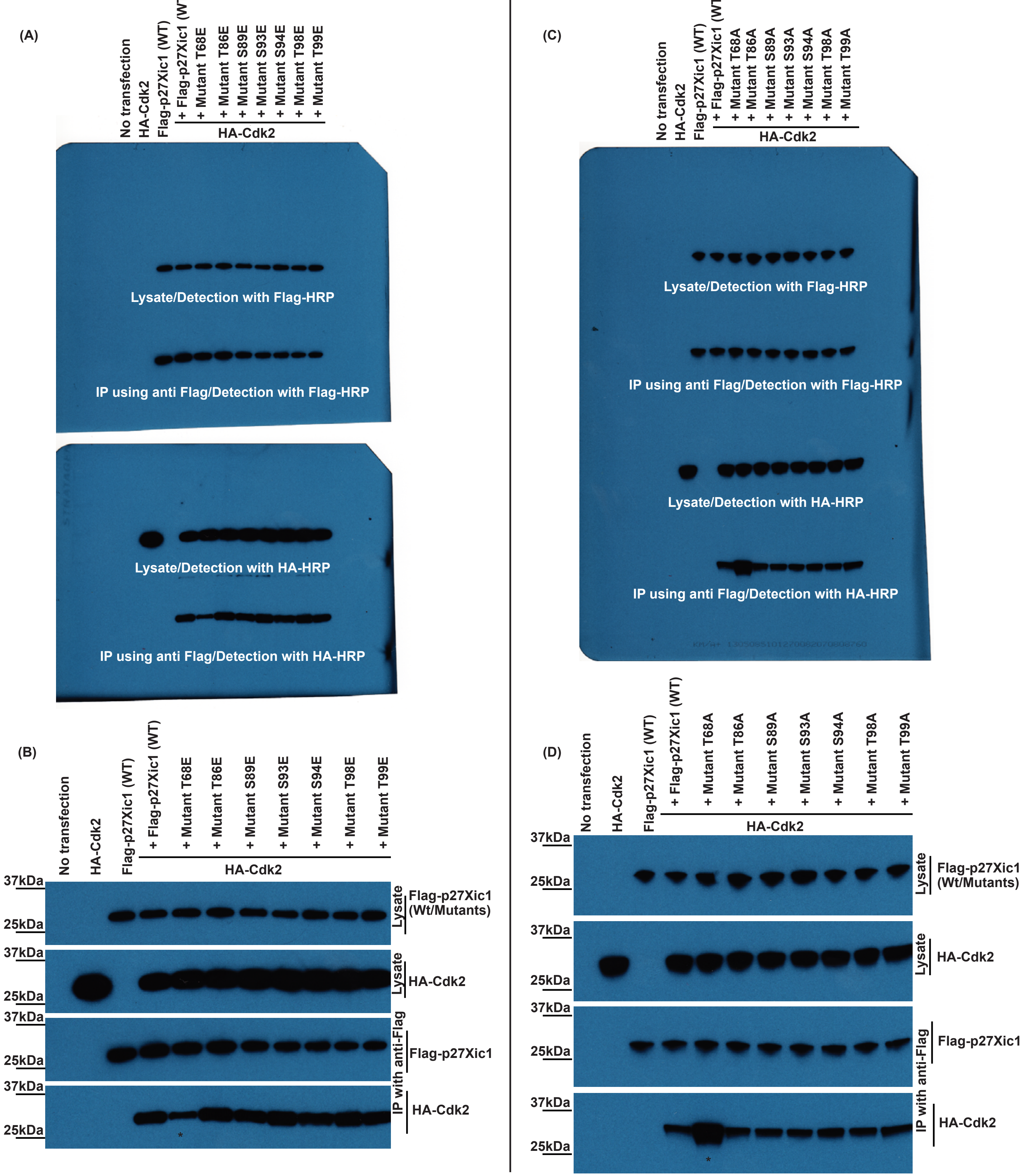


**FigS5, related to fig4C and D:** Blots on the left hand side show raw data (above) and processed data (below) for fig4C, while blots on the right hand side show raw data (above) and processed data (below) for fig4D.

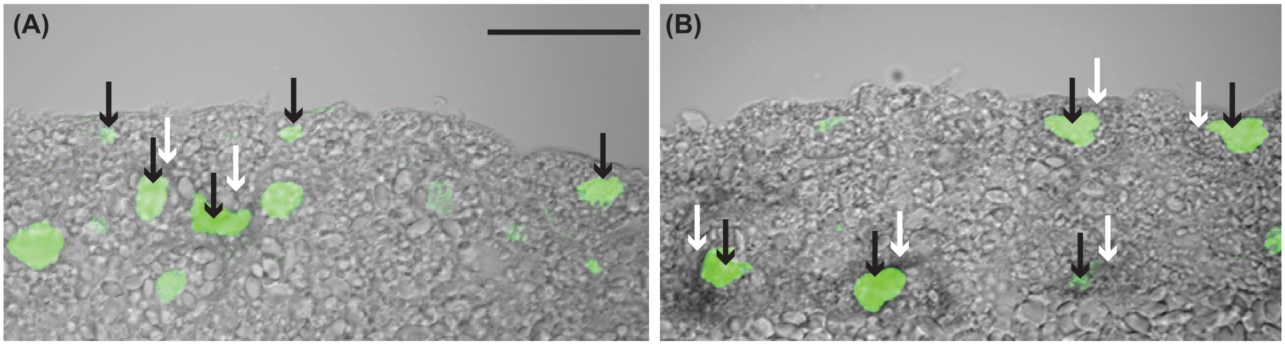


**FigS6, related to fig5: (A)** p27Xic1 promotes neurogenesis in a cell autonomous manner. To see if the effects of p27Xic1 on neurogenesis were cell autonomous or not, we combined the in situ hybridisation (non-fluorescent, black precipitation) against neuronal differentiation marker *N-tubulin* with immunostaining (fluorescent, green) against Flag-p27Xic1 using anti-Flag antibody, on embryos overexpressing Flag-p27Xic1. Sections from the embryos were imaged. As an example of the sections shows, overexpression of Flag-p27Xic1 (green, black arrow) promotes neurogenesis (*N-tubulin*, black precipitation, white arrow) within the same cells overexpressing p27xic1. In image 'A', outer superficial polarised cells show no *N-tubulin* expression even after Flag-p27Xic1 overexpression. **(B)** Phospho-mutant p27Xic1 can also promote neurogenesis in the outer superficial polarized cells as well. Cells overexpressing Phospho-mutant Flag-p27Xic1 (that can not be rescued by aPKC unlike wild type Flag-p27Xic1) promotes neurogenesis not only in the inner nonpolar cells but in the outer superficial polarised cells as well. The images also show that Flag-p27Xic1 protein is localised exclusively to the cell nucleus. Black scale bar in A represents 50μM.

## Legends for supplemental movies

**Movie S1A, related to fig2B – Time-lapse movie for HeLa Fucci biosensor treated with DMSO (control).** Movie shows HeLa Fucci cells dividing in the presence of DMSO (control). Time-lapse images were analyzed and converted into AVI movies using ImageJ software. Appearance of an arrow marks the dividing cells in the movies. Cell cycle parameters were calculated as explained in supplemental experimental procedure 1. Please refer to fig2B for calculated parameters.

**Movie S1B, related to fig2B – Time-lapse movie for HeLa Fucci biosensor treated with aPKC specific myristoylated pseudosubstrate inhibitor.** Movie shows HeLa Fucci cells dividing in the presence of aPKC specific inhibitor (Myristoylated pseudosubstrate inhibitor/Myr PSI). Time-lapse images were analyzed and converted into AVI movies using ImageJ software. Appearance of an arrow marks the dividing cells in the movies. Cell cycle parameters were calculated as explained in supplemental experimental procedure 1. Please refer to fig2B for calculated parameters. The movie shows that on aPKC inhibition, Fucci cells exhibit longer cell cycle length (Tc) due to longer G1 phase length ($T_{G1}$) and longer S phase length ($T_S$).

**Movie S1C, related to fig2B – Time-lapse movie for HeLa Fucci biosensor treated with aPKC inhibitor (GÖ6983/GO).** Movie shows HeLa Fucci cells dividing in the presence of aPKC inhibitor (GÖ6983/GO). Time-lapse images were analyzed and converted into AVI movies using ImageJ software. Appearance of an arrow marks the dividing cells in the movies. Cell cycle parameters were calculated as explained in supplemental experimental procedure 1. Please refer to fig2B for

calculated parameters. The movie shows that on aPKC inhibition, Fucci cells exhibit longer cell cycle length (Tc) due to longer G1 phase length ($T_{G1}$) and longer S phase length ($T_S$).

## Supplemental experimental procedure

**1. Estimation of cell cycle kinetics parameters using DPSL, PLM and Fucci biosensor analysis** DPSL was applied to embryos to estimate total cell cycle length ($T_C$) and S-phase length ($T_S$), for polar and nonpolar Sox3+ progenitors and for injected vs noninjected sides (Fig1Ac, 2Ac). PLM performed for the duration of 240 mins gave us an estimate of the length of G2+1/2M-phases (Fig1Bc). Mitotic index information in combination with $T_C$ estimated by DPSL was used to estimate $T_M$ (time for mitosis) for both progenitors (Fig1Bd). $T_M$ for polar progenitors calculated this way matched with the $T_M$ calculated by live imaging (data not shown). Estimated $T_M$ values in combination with the length of G2+1/2M gave estimation for the length of G2. Finally, length of G1 phase was calculated by deducting lengths of S, G2 and M phases from the length of the total cell cycle [$T_{G1}=T_C-(T_S+T_M+T_{G2})$].

For the purpose of estimating cell cycle kinetics parameters on cells, HeLa Fucci live cells were imaged as described before (Sakaue-Sawano et al., 2008). Fucci cells can be used visually to calculate the exact length of different cell cycle phases (G1 with Red fluorescence, early S with Yellow fluorescence, SG2M with Green fluorescence and M with round shaped cells, schematic in Fig2B) in control and experimental condition. Roughly 120cells/well were plated on a 96-well glass-bottom dish on day0. On day1, normal DMEM media was replaced with movie media (Nutrient Mixture F-12 Ham, Sigma Aldrich) containing appropriate amount of chemical inhibitor. On day2, control and experimental cells were imaged on a heated stage at 37$^0$C and 5% CO2 supply using NikonA1 confocal microscope for 2-3 days. Images were captured every 20 mins. Collected images were converted into time-lapse movies and analyzed using ImageJ software, for estimating the lengths of various cell cycle phases.

**2. Plasmid constructs** Constructs relating to aPKC are already described (Sabherwal et al., 2009). For the p27Xic1 constructs (pGEX-6P-GST-p27Xic1, pCS2-3Flag-p27Xic1 and deletion constructs used in immuno-complex kinase assays), desired coding regions were PCR-amplified using clone TTpA019B19 from neurula stage *Xenopus tropicalis* EST library as template and cloned into appropriate vectors. Phospho- and phospho-mimetic mutants against the sites identified by mass-spectrometry analysis of p27Xic1 protein post *in vitro*- and *in vivo*-kinase assays were generated using Quick Change site-directed mutagenesis kit (Agilent Technologies), following manufacturer's guidelines. Full-length coding regions for Cdk2 and Cdk4 were PCR-amplified using clones TNeu043b09 and TNeu018k23, respectively, from neurula stage *X. tropicalis* library and cloned into pCS2-HA expression vector.

**3. Protein half-life assay** HeLa cells overexpressing p27Xic1 alone or in combination with aPKC-CAAX were treated with 100µM Cycloheximide (Sigma Aldrich) for the designated time periods and protein half-life assays were performed as described before (Scott, Ingram, & Ball, 2002). Please refer to figS3 for data.

**4. Sample preparation for mass-spectrometry analysis** To prepare samples for MS analysis, HeLa cells grown to 80-90% confluency in 10cm cell culture dishes were transfected with Flag-p27Xic1 with HA-aPKC-CAAX (for *in vivo* kinase sample), or alone (for control for *in vivo* kinase sample, or for *in vitro* kinase reaction). 24hrs post transfection, cells were harvested and lysed in 1ml of lysis buffer containing protease and phosphatase inhibitors (Thermo Scientific). Flag-p27Xic1 was immunoprecipitated (IPed) as described in materials and methods, using 25µl of ProteinA/G beads and 100µg of anti-Flag antibody. For *in vitro* kinase reaction, IPed Flag-p27Xic1 was mixed with 1µg of His-aPKC (Millipore) and cold ATP as

described in materials and methods. IPed Flag-p27Xic1 was run on 10% gel. Post run, gel was stained using Coomassie based InstantBlue solution (Expedeon) following manufacturer's instructions.
