# Supplementary material for: aPKC Phosphorylates p27Xic1, Providing a Mechanistic Link between Apicobasal Polarity and Cell-Cycle Control
Source: Dev Cell. 2014 Dec 8;31(5):559–71. doi: 10.1016/j.devcel.2014.10.023 (PMC4262734; doi:10.1016/j.devcel.2014.10.023)
Supplement: Document S2. Article plus Supplemental Information [file mmc3.pdf]

Developmental Cell
Article

Cell Press

aPKC Phosphorylates p27Xic1, Providing a Mechanistic Link between Apicobasal Polarity and Cell-Cycle Control

Nitin Sabherwal,[1,*] Raphael Thuret,[1] Robert Lea,[1] Peter Stanley,[1] and Nancy Papalopulu[1,*]
[1]Faculty of Life Sciences, University of Manchester, Oxford Road, Manchester M13 9PT, UK
*Correspondence: nitin.sabherwal@manchester.ac.uk (N.S.), nancy.papalopulu@manchester.ac.uk (N.P.)
http://dx.doi.org/10.1016/j.devcel.2014.10.023
This is an open access article under the CC BY license (http://creativecommons.org/licenses/by/3.0/).

SUMMARY

During the development of the nervous system, apicobasally polarized stem cells are characterized by a shorter cell cycle than nonpolar progenitors, leading to a lower differentiation potential of these cells. However, how polarization might be directly linked to the kinetics of the cell cycle is not understood. Here, we report that apicobasally polarized neuroepithelial cells in *Xenopus laevis* have a shorter cell cycle than nonpolar progenitors, consistent with mammalian systems. We show that the apically localized serine/threonine kinase aPKC directly phosphorylates an N-terminal site of the cell-cycle inhibitor p27Xic1 and reduces its ability to inhibit the cyclin-dependent kinase 2 (Cdk2), leading to shortening of G1 and S phases. Overexpression of activated aPKC blocks the neuronal differentiation-promoting activity of p27Xic1. These findings provide a direct mechanistic link between apicobasal polarity and the cell cycle, which may explain how proliferation is favored over differentiation in polarized neural stem cells.

INTRODUCTION

The cell cycle is a fundamental cellular process that governs the ability of cells to divide. Control of the cell cycle is crucial for the generation of tissues from dividing stem cells, such as the development of the nervous system. Exit from the cell cycle is associated with the control of differentiation because differentiated cells tend to be postmitotic. Re-entry of differentiated neurons into the cell cycle leads to apoptosis (Folch et al., 2012), although there are some examples where the cells have been shown to differentiate even if cell-cycle exit is prevented (Lobjois et al., 2008). Recently, not only cell-cycle exit, but also the length of the cell cycle, in particular the G1 phase length, has been associated with the decision of cells to differentiate. During mouse cortical development, elongation of the G1 phase by inhibiting the G1 cyclin-dependent kinases (Cdk4/6) promotes neurogenesis, whereas shortening of G1 by overexpressing G1 kinase Cdk4/CyD1 promotes proliferative divisions (Lange and Calegari, 2010; Lange et al., 2009). A correlation between cell cycle/G1 length and the propensity for differentiation has also been documented in embryonic stem cells (Roccio et al., 2013; Coronado et al., 2013) and neural progenitors in the chicken spinal cord (Wilcock et al., 2007). Apart from G1, the other two phases of interphase, G2 and S, have also been linked to neuronal differentiation. Expanding progenitors in the mouse cortex have been shown to have a longer S phase (Arai et al., 2011) and elongation of the G2 phase has also been shown to promote progenitor proliferation (Peco et al., 2012), although in the latter case, the effect has been linked back to the shortening of the G1 phase. This is not surprising because G1 is the key cell-cycle phase where both intrinsic and extrinsic signaling pathways impinge to instruct the cell whether to go for another round of division or differentiate (Hindley and Philpott, 2012).

Why do some progenitors have a longer cell-cycle/G1 phase? An intriguing observation is that in the cortex, progenitors with a longer G1 phase are nonpolar (basal progenitors) whereas progenitors with shorter G1 are apicobasally polarized (apical progenitors; Arai et al., 2011). It is also known that in many systems such as *Drosophila* neuroblasts, and mouse, *Xenopus*, and chicken neuroepithelium, polarized progenitors have a lower propensity to differentiate than their nonpolar daughter cells (Sabherwal and Papalopulu, 2012). These results point to a link between polarity and the cell cycle, however, a direct mechanistic link between these two biological processes is not known. Any such mechanism is likely to involve key regulators of cell-cycle progression, such as the cyclin/cyclin-dependent kinase (Cy/Cdk) complexes and their inhibitors (cyclin-dependent kinase inhibitors/CdkIs). Inhibition of Cdk activity by stronger association with CdkIs has been shown to trigger neuronal differentiation (Kranenburg et al., 1995). Conversely, loss of CdkIs has been shown to inhibit differentiation (Carruthers et al., 2003; Mairet-Coello et al., 2012; Vernon et al., 2003).

Here, we have used the neuroectoderm of the *Xenopus* embryo to investigate how the apicobasal polarization of neural progenitor cells affects cell-cycle kinetics and consequently, neuronal differentiation. *Xenopus* neuroectoderm exhibits a clean segregation of polarized and nonpolar progenitors into two distinct layers and provides a simple and accessible model for answering such fundamental questions. In *Xenopus*, deep (inner) nonpolar cells have a high propensity to differentiate, whereas superficial (outer) apicobasally polarized neuroepithelial cells are intrinsically resistant to primary neuronal differentiation (Chalmers et al., 2002). Using this system, we have previously shown that the key regulator of polarity, the atypical

(serine-threonine) protein kinase C (aPKC) has an instructive role in promoting proliferation and suppressing differentiation in polarized progenitors (Sabherwal et al., 2009). In this paper, we show that, first, the cell-cycle kinetics of polarized and nonpolar neuroectodermal cells differ significantly, with polarized cells having shorter total cell-cycle length and shorter S and G1 phases than nonpolar cells. Overexpression of p27Xic1, a member of the CIP/KIP CdkI family, elongates the G1 phase of the cell cycle and promotes terminal differentiation during *Xenopus* primary neurogenesis, a phenotype opposite to that of overexpression of an activated membrane-targeted form of aPKC, aPKC-CAAX. aPKC-CAAX overexpression rescues the increased neuronal differentiation phenotype of p27Xic1 overexpression, as would be expected if aPKC counteracted the activity of p27Xic1. Then, we show that aPKC directly phosphorylates p27Xic1 in the N terminus of the protein. Phosphomimetic p27Xic1 shows reduced binding to the G1-S related cyclin-dependent kinase 2 (Cdk2), resulting in reduced inhibition and higher kinase activity, which in turn causes a faster cell cycle. This study thus identifies a direct mechanistic link between apicobasal cell polarity and the cell cycle.

RESULTS

Polarized and Nonpolar Neural Progenitors in *Xenopus* Neuroectoderm Have Different Cell-Cycle Kinetics

Dual-pulse S phase labeling (DPSL) analysis on outer layer apicobasally polarized progenitors and inner layer nonpolar neural progenitors (both Sox3+), showed that polarized progenitors have a significantly shorter cell cycle length and a shorter S phase length than nonpolar progenitors at open neural plate stage, NF13 (T_C, polarized versus nonpolar, mean ± SEM, 282 ± 14 min versus 411 ± 21 min, and $T_{S,}$ polarized versus nonpolar, 40 ± 4 min versus 141 ± 14 min, Figure 1A). We also analyzed these progenitors for percentage of labeled mitoses (PLM) to estimate G2+1/2M phase length and compared them for their mitotic indices (Figure 1B). Putting the numbers together from these experiments (see Supplemental Experimental Procedures available online for details) showed that polarized progenitors have a shorter G1 phase (T_{G1}, 94 min versus 160 min for nonpolar cells) but a longer G2 phase than the nonpolar progenitors (T_{G2}, 132 min versus 89 min). The lengths of mitoses were marginally different between the two layers (T_M, 16 min versus 21 min) (Figure 1C). Thus, establishing the cell-cycle kinetic parameters for polarized and nonpolar progenitors showed that they differ significantly and that polarized progenitors cycle faster.

aPKC Promotes Neural Progenitor Proliferation and Affects Cell Cycle Length

Confirming our previous findings (Sabherwal et al., 2009), overexpression of constitutively active, membrane-targeted aPKC (aPKC-CAAX) suppressed neuronal differentiation, as judged by the reduction in the expression of a terminal differentiation marker *N-tubulin*. Conversely, nuclear dominant-negative aPKC (NLS-aPKC-NT) promoted neuronal differentiation as *N-tubulin* was enhanced (Figure S1 available online). Extending these observations further, we found that on aPKC-CAAX overexpression, *ElrC*, a marker of committed neural progenitors (Carruthers et al., 2003), was also suppressed, whereas cells expressing the neural progenitor marker *Sox3* showed expansion (*Sox3*+ area was increased on the injected side as shown by in situ hybridization) (Figure S1). This suggested that aPKC-CAAX suppressed neuronal differentiation by promoting neural progenitor expansion. In aPKC-CAAX-overexpressing embryos, the number of Sox3+ progenitors on the injected side was significantly higher (shown by immunostaining, Figure 2A) and cells on the injected side had significantly shorter T_C and T_S, than on the noninjected side, calculated by the DPSL technique. Control embryos overexpressing GFP-CAAX exhibited no such differences (Figure 2A).

To further see the effects of aPKC on different phases of the cell cycle, we treated and imaged HeLa Fucci cells (Sakaue-Sawano et al., 2008) for 48–60 hr in the presence of a myristoylated, cell-permeable inhibitor specific against aPKC (Sajan et al., 1999). Cells inhibited for aPKC showed significant lengthening of the total cell-cycle time (T_C, 25.32 ± 0.58 hr versus 19.38 ± 0.45 hr for controls). This was mainly attributed to the lengthening of the G1 phase (T_{G1}, 17.04 ± 0.47 hr versus 11.79 ± 0.51 hr for controls), with small effects observed in the early S phase (T_S, 5.68 ± 0.78 hr than 3.38 ± 0.40 hr for controls). M phase length (T_M) was not affected by the treatment. The effect on the length of S, G2, and M together (T_{SG2M}, 14.15 ± 0.67 hr versus 10.92 ± 0.43 hr for controls) indicated that the G2 phase was also largely unaffected by the inhibitor treatment (Figure 2B and Movies S1A and S1B).

Pseudosubstrate inhibitors against kinases have the highest specificity. The pseudosubstrate myristoylated inhibitor used here is highly specific and has no effect on classical and novel PKCs (Standaert et al., 1999). Nevertheless, these data were further substantiated by the experiment with a chemical inhibitor against aPKC, bisindolylmaleimide Gö6983. This inhibitor has been used previously to show that aPKC is involved in TE formation (Eckert et al., 2004) and can maintain ES cells in an undifferentiated state in the absence of LIF, through the inhibition of PKCζ (Dutta et al., 2011). Gö6983 showed similar effects on cell-cycle kinetics as shown by Myr inhibitor against aPKC (Figure 2B and Movies S1A and S1C).

The Fucci data along with DPSL data suggested that both gain and loss of aPKC signaling activity affects cell cycle length via G1 and S phases, with G2 and M phases remaining largely unaffected. These data suggested that the effects of aPKC on progenitors' proliferation might be mediated via its effects on cell-cycle kinetics. To test this further, we investigated the interaction of aPKC with cell-cycle regulators.

aPKC Directly Phosphorylates CIP/KIP Cell-Cycle Inhibitor p27Xic1 Both In Vitro and In Vivo

G1 kinases (Cdk4/6) and G1/S kinase (Cdk2) are positively regulated by CyD and CyE/A, respectively, and negatively regulated by a CIP/KIP family cyclin-dependent kinase inhibitor (CdkI) p27Xic1 (Ohnuma and Harris, 2003). Because cyclins D, E, A, and p27Xic1 are regulated by posttranslational modifications, mainly phosphorylations (Alberts et al., 2002), we analyzed them for being phosphorylation targets of aPKC. In vitro kinase assays showed that only bacterially expressed p27Xic1 is a direct phosphorylation target of GST-aPKC (commercially supplied) (Figure 3A and data not shown). The specificity of this in vitro phosphorylation was confirmed by performing the kinase assay in the presence of a pseudosubstrate inhibitor specific

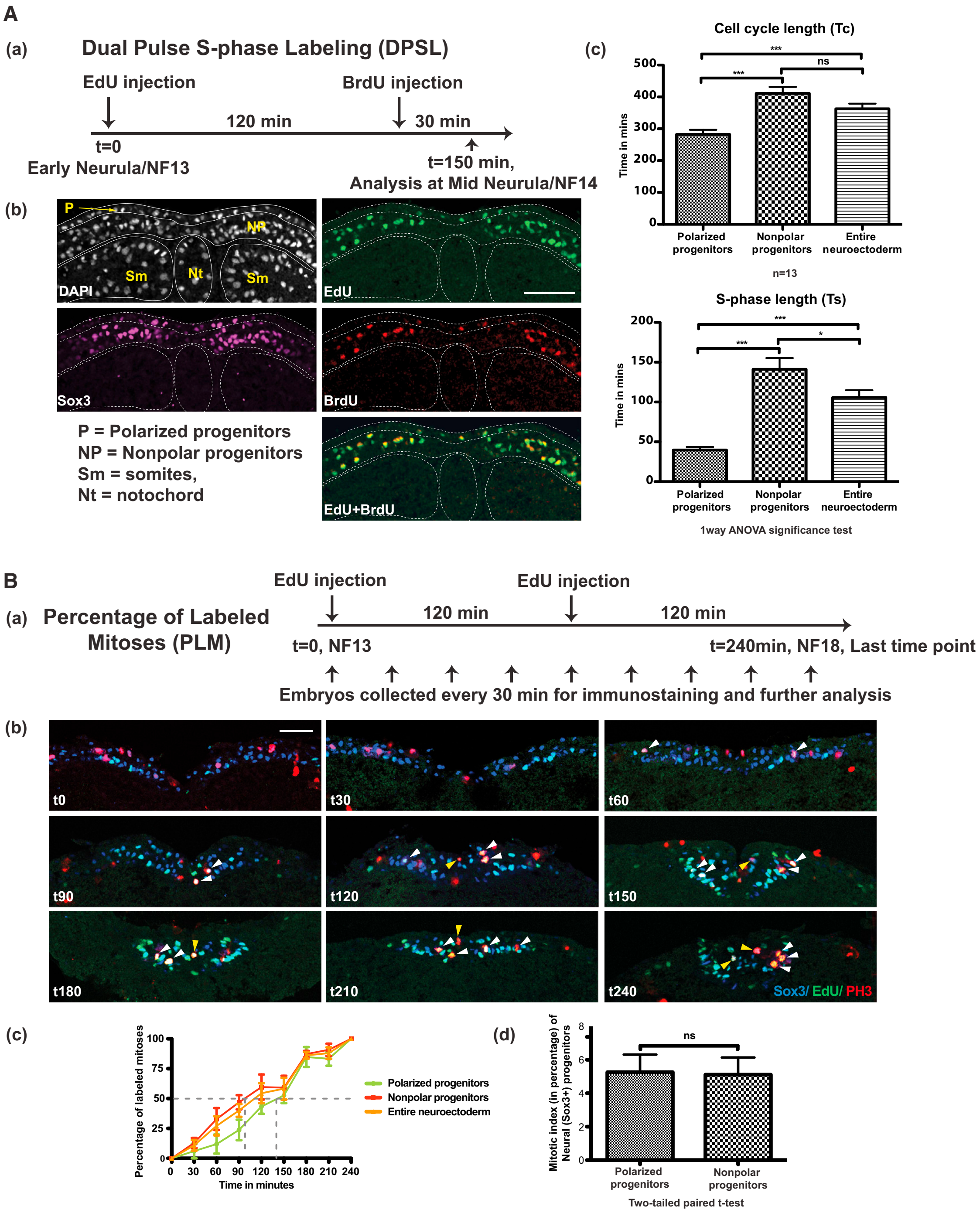

C

Durations in mins	Polarized progenitors	Nonpolar progenitors	Source
T_C	282	411	DPSL
T_S	40	141	DPSL
$T_{G2+M+G1}$	242	270	DPSL
T_M	16	21	PLM
T_{G2}	132	89	PLM
T_{G1}	94	160	DPSL+PLM

Figure 1. Polarized and Nonpolar Neural Progenitors Have Significantly Different Cell-Cycle Kinetics Parameters

(A) Dual pulse S phase labeling (DPSL) technique applied on wild-type NF13 *Xenopus* embryos (a) and analyzed by sectioning (b) shows that outer polar progenitors have shorter cell cycle (T_C) and S phase (T_S) lengths than inner nonpolar progenitors (c, mean ± SEM).

(B) Similar embryos were processed for percentage of labeled mitoses (PLM, to estimate $T_{G2+1/2M}$) (a–c). Representative sections are shown in (b), in which white arrowheads show labeled mitoses for nonpolar progenitors, while yellow arrowheads show labeled mitoses for polarized progenitors. During the time course of

(legend continued on next page)

against aPKC (Sajan et al., 1999). This reduced the kinase signal in a dose-dependent manner (Figure 3B). To see if aPKC can phosphorylate p27Xic1 in a complex embryonic milieu as well, we performed in vitro kinase assay using embryonic lysate, instead of kinase buffer, as the medium for interaction between exogenous GST-p27Xic1 (bacterially expressed) and His-aPKC (commercially supplied). The result demonstrated that phosphorylated GST-p27Xic1 significantly increases in the presence of aPKC, whereas it drops down to basal level in the presence of pseudosubstrate inhibitor against aPKC (Figure 3C).

To further substantiate these observations in vivo, we performed in vivo kinase assay measuring the incorporation of γ-P32-labeled orthophosphate by p27Xic1, overexpressed in HeLa cells along with aPKC-CAAX or in the presence of myristoylated pseudosubstrate aPKC inhibitor. The experiment showed that p27Xic1 incorporated more orthophosphate when expressed along with aPKC-CAAX; the incorporation was reduced when the same cells were cultured in the presence of the inhibitor (Figure 3D). p27Xic1 and aPKC-CAAX also showed direct physical interaction in coimmunoprecipitation (coIP) assays. First, Flag-p27Xic1 and HA-aPKC-CAAX constructs were co-overexpressed in HeLa cells and HA-aPKC-CAAX was detected after IP of Flag-p27Xic1 using anti-Flag beads (Figure 3E). More importantly, the endogenous p27Xic1 and aPKC from embryonic lysate also showed direct physical interaction in coIP experiments where either p27Xic1 or aPKC is immunoprecipitated with antibodies and aPKC or p27Xic1 is detected by western blot in the pull-down, respectively (Figures 3F and S2).

The effect of aPKC inhibitors on HeLa Fucci cell-cycle length (Figure 2B) suggested that human p27Kip1 might also be negatively regulated by aPKC and may be a phosphorylation target of aPKC. However, our in vitro kinase assay using immunoprecipitated p27Kip1 from HeLa cells showed that human p27Kip1 is not a direct phosphorylation target of aPKC (data not shown). This suggests that aPKC negatively regulates p27Kip1 in an indirect manner.

Regulation of p27Xic1 Activity by aPKC Likely Takes Place in the Cell Nucleus

To gain some insight into where the interaction of aPKC and p27Xic1 may be taking place, we performed immunostaining on neurula stage embryos overexpressing Flag-p27Xic1 and HA-aPKC-CAAX. These showed that p27Xic1 was largely in the nucleus of the cells, and a portion of aPKC-CAAX, which was mostly localized to the cell cortex, was also found in the nucleus (Figure S3). These findings are consistent with previous reports showing nuclear enrichment of p27Xic1 (Chuang and Yew, 2001; Chuang et al., 2005) and some nuclear localization of aPKC-CAAX (Sabherwal et al., 2009) and lend support to the idea that aPKC interacts with p27Xic1 in the nucleus. In our previous publication (Sabherwal et al., 2009), we showed that aPKC-CAAX is more active and nuclear than aPKC and hypothesized that after getting activated in the membrane, a small proportion of aPKC-CAAX is translocated to the nucleus. This suggests that although aPKC-CAAX is predominantly membrane localized and weakly nuclear, it is highly active and sufficient to influence nuclear events like interacting with p27Xic1 and modulating its activity.

aPKC Phosphorylates p27Xic1 in Its Cdk-Interaction Domain, Resulting in Its Reduced Binding to Cdks

To identify domains of p27Xic1 phosphorylated by aPKC, we performed immunocomplex kinase assays on Flag-tagged deletion constructs of p27Xic1. As shown in Figure 4A, the N-terminal and middle parts of the protein showed positive kinase signal whereas the C-terminal fragment showed no sign of phosphorylation during in vitro kinase assays. Phosphosite identification using liquid chromatography/tandem mass spectrometry (LC/MS/MS) analysis on Flag-p27Xic1 phosphorylated by aPKC in vitro (i.e., in vitro kinase reaction on immunoprecipitated/IPed Flag-p27Xic1 from HeLa cells) identified multiple phosphorylation sites with significant Ascore values ($\geq$13, Ascore is a measurement of the confidence of phosphorylation; Beausoleil et al., 2006) within the N-terminal, Cdk-binding domain (Figure 4A). To see if the sites identified correspond to the phosphosites in vivo, similar LC/MS/MS analysis was carried out on Flag-p27Xic1 IPed from HeLa cells co-overexpressing it with HA-aPKC-CAAX. This analysis identified a phosphosite (T68) with a significantly high Ascore (Ascore = 27; Figure 4A), located within the Cdk interaction domain of p27Xic1; another site (T99, located immediately after the Cdk interaction domain) was picked with a low Ascore value (Ascore = 13; Figure 4A). None of these sites was identified on Flag-p27Xic1 IPed from HeLa cells overexpressing it alone. *Xenopus* p27Xic1 is 44% identical to human p27Kip1 and 40% identical to human p21Cip1 in the conserved N terminus. It also possesses a PCNA-like binding site characteristic of p21Cip1 in the C terminus (Su et al., 1995). The aPKC phosphosite in p27Xic1 (T68A) is not conserved in mammalian p27Kip1 but is conserved in mammalian p21Cip1 (although it has not been identified as an aPKC phosphosite by others) and p27Xic1 from zebrafish (Figure 4B), suggesting some degree of functional conservation linking aPKC activity and cell-cycle regulation; the other phosphosite identified (T99) shows no conservation with other members of CIP/KIP CdkIs.

Identification of aPKC phosphorylation sites prompted us to check if these phosphorylation events are functionally important by generating phospho (S/T to A) or phosphomimetic (S/T to E) mutants. Initial protein abundance experiments showed that the level of Flag-p27Xic1 protein is decreased in embryos injected with HA-aPKC-CAAX and increased in embryos injected with the dominant-negative HA-aPKC-NT (Figure S4A). Protein stability experiments using cycloheximide showed that the half-life of overexpressed Flag-p27Xic1 was reduced when it was co-overexpressed in HeLa cells along with HA-aPKC-CAAX (Figure S4B). However, nonphosphorylatable mutants (like T68A) were also destabilized in comparison to the wild-type protein, whereas phosphomimetic mutants (like T68E) of p27Xic1 appeared more stable (see Figures S4C and S4D for

the experiment, neural plate cells converge to the midline. Percentage of mitotic indices for polarized and nonpolar progenitors showed no differences (d, mean ± SEM).

(C) Table summarizes different cell-cycle parameters for the two types of progenitors. See the Experimental Procedures and Supplemental Experimental Procedures for details about the techniques and calculations of kinetic parameters. All scale bars represent 100 μM.

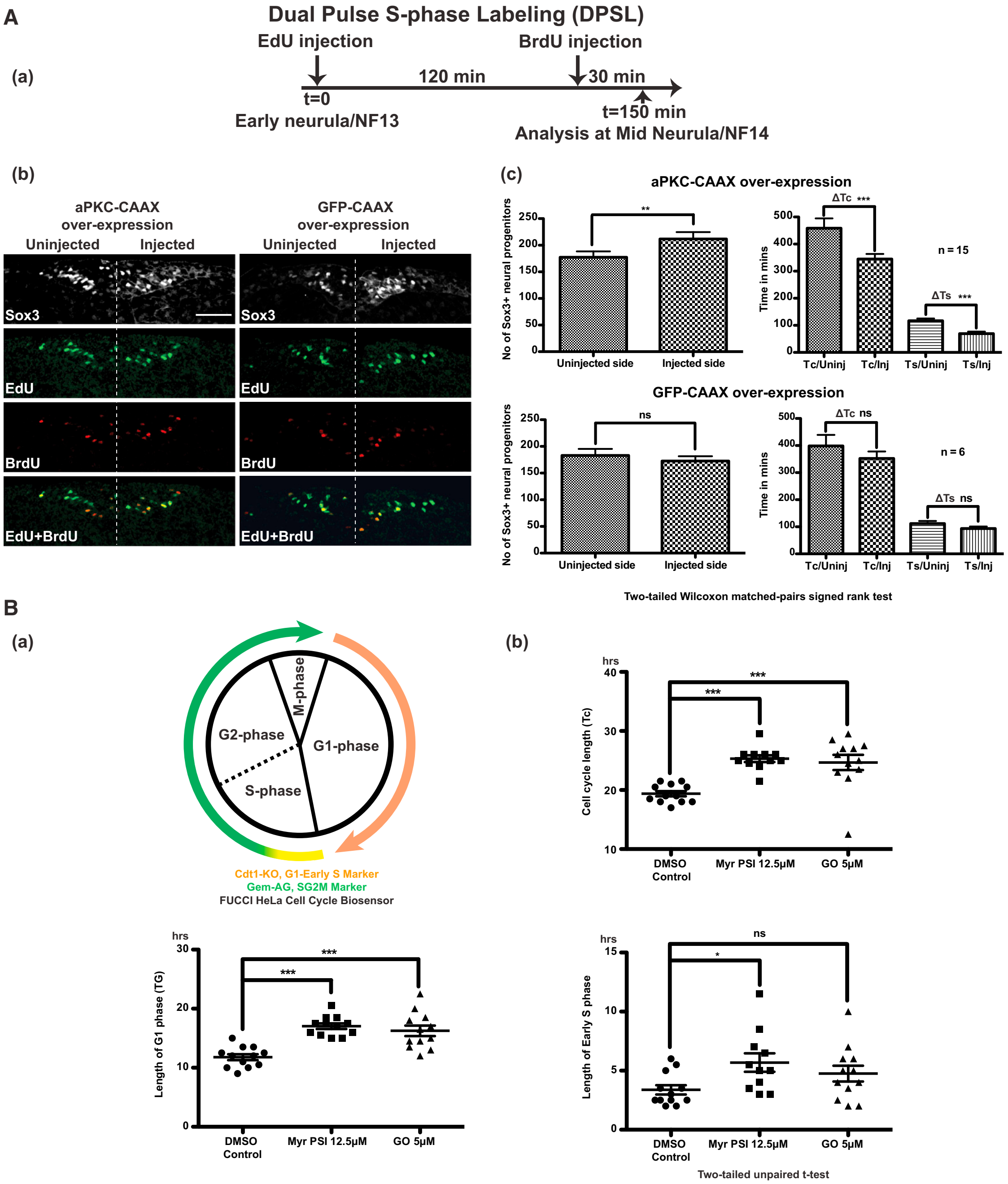

Figure 2. Overexpressing Activated aPKC Causes Progenitor Proliferation and Shortens the Cell Cycle by Shortening G1 and S Phases

(A) DPSL analysis (a) shows that embryos on the aPKC-CAAX injected side show higher number of Sox3+ progenitors (b and c) and have shorter cell cycle (T_C) and S phase (T_S) (c, mean ± SEM). Control embryos overexpressing GFP-CAAX showed no such differences. The scale bar in (Ab) represents 100 μM. See also Figure S1.

(B) Schematic of Fucci cell cycle biosensor, which can be visually used to measure the lengths of various phases of cell cycle, is shown (a). When live imaged in the presence of either a pseudosubstrate inhibitor (MyrPSI) or a chemical inhibitor (Gö6983/GO) against aPKC, HeLa Fucci cells showed a significant increase in cell-cycle length (T_C) due to elongated G1 and early S phases (b, mean ± SEM). Time-lapse movies are shown in Movies S1A–S1C.

examples), suggesting that the effect of aPKC phosphorylation on p27Xic1 stability is complex and cannot be reproduced by single amino acid changes.

Not only the stability of CdkIs, but also the inhibition of Cdk kinase activity by CdkIs has been shown to promote differentiation (Hasan et al., 2013). We tested whether the binding of p27Xic1 to Cdks is affected by aPKC phosphorylation, which could explain the shortening of G1 and S phases of cell cycle. p27Xic1 interacts with Cdk2 and Cdk4 and inhibits their kinase activities (Finkielstein et al., 2001). CoIP assays using HeLa cells

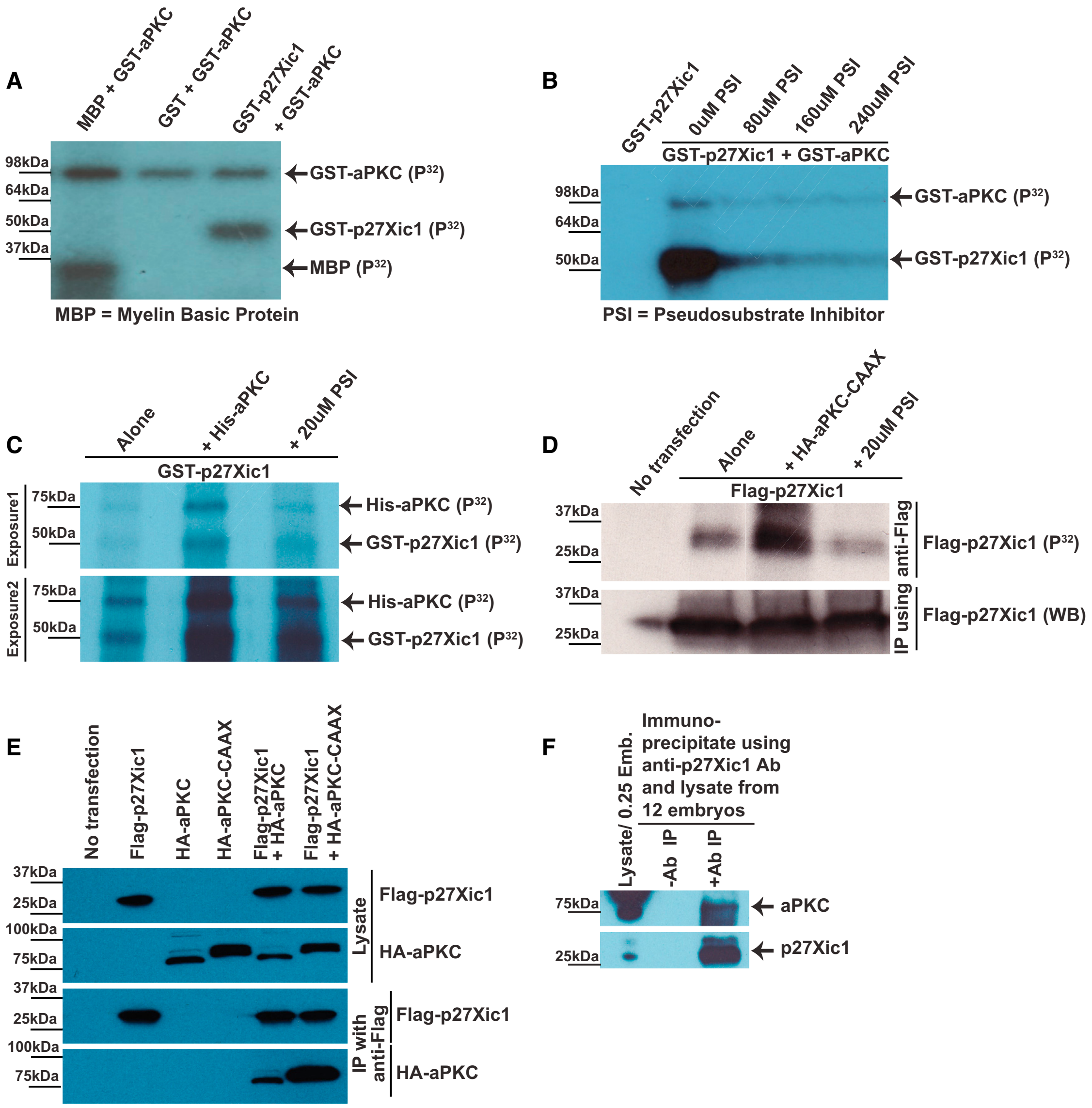

Figure 3. aPKC Directly Phosphorylates p27Xic1

(A) In vitro kinase assays showing that aPKC phosphorylates bacterially expressed GST-p27Xic1. Myelin basic protein (MBP) was used as a positive marker for aPKC phosphorylation.

(B) Specificity of the kinase reaction was confirmed by using a pseudosubstrate inhibitor of aPKC.

(C) Kinase assay performed in an embryonic environment (using embryo lysate instead of kinase buffer) also confirmed p27Xic1 being a direct phosphorylation target of aPKC.

(D) In vivo kinase assay shows that p27Xic1 incorporates higher amount of P32 when it is co-overexpressed along with aPKC-CAAX; the amount of incorporated P32 is lower when p27Xic1 in overexpressed in HeLa cells in the presence of pseudosubstrate inhibitor against aPKC.

(E) CoIP of HA-aPKC-CAAX with Flag-p27Xic1 in HeLa cells shows that aPKC-CAAX and p27Xic1 interact physically.

(F) Physical interaction between p27Xic1 and aPKC was confirmed for endogenous proteins from embryonic lysates by a coIP assay using antibodies against endogenous p27Xic1 and aPKC. See also Figures S2 and S3.

co-overexpressing Flag-p27Xic1 along with either HA-Cdk2 or HA-Cdk4 showed that p27Xic1 binds to Cdk2 much stronger than to Cdk4 (data not shown). Similar coIP assays showed that phosphomimetic mutant T68E showed almost 50% reduction in its binding to Cdk2 in comparison to the wild-type p27Xic1 (Figures 4C, 4E, and S5), whereas the phosphomutant T68A showed enhanced binding (increased by almost 140%) to Cdk2 (Figures 4D, 4E, and S5). Other mutants showed binding similar to the wild-type p27Xic1 (Figures 4C, 4D, and S5). These assays suggested that a single phosphorylation event at amino acid T68 of p27Xic1 by aPKC is enough to affect its interaction with Cdk2. Using Histone H1 as a substrate for active Cdk2/CyclinA, we found that in comparison to the wild-type p27Xic1, phosphomutant T68A abolished the kinase activity of Cdk2 almost completely, while the phosphomimetic mutant T68E had a negligible effect on Cdk2 activity in the same assay

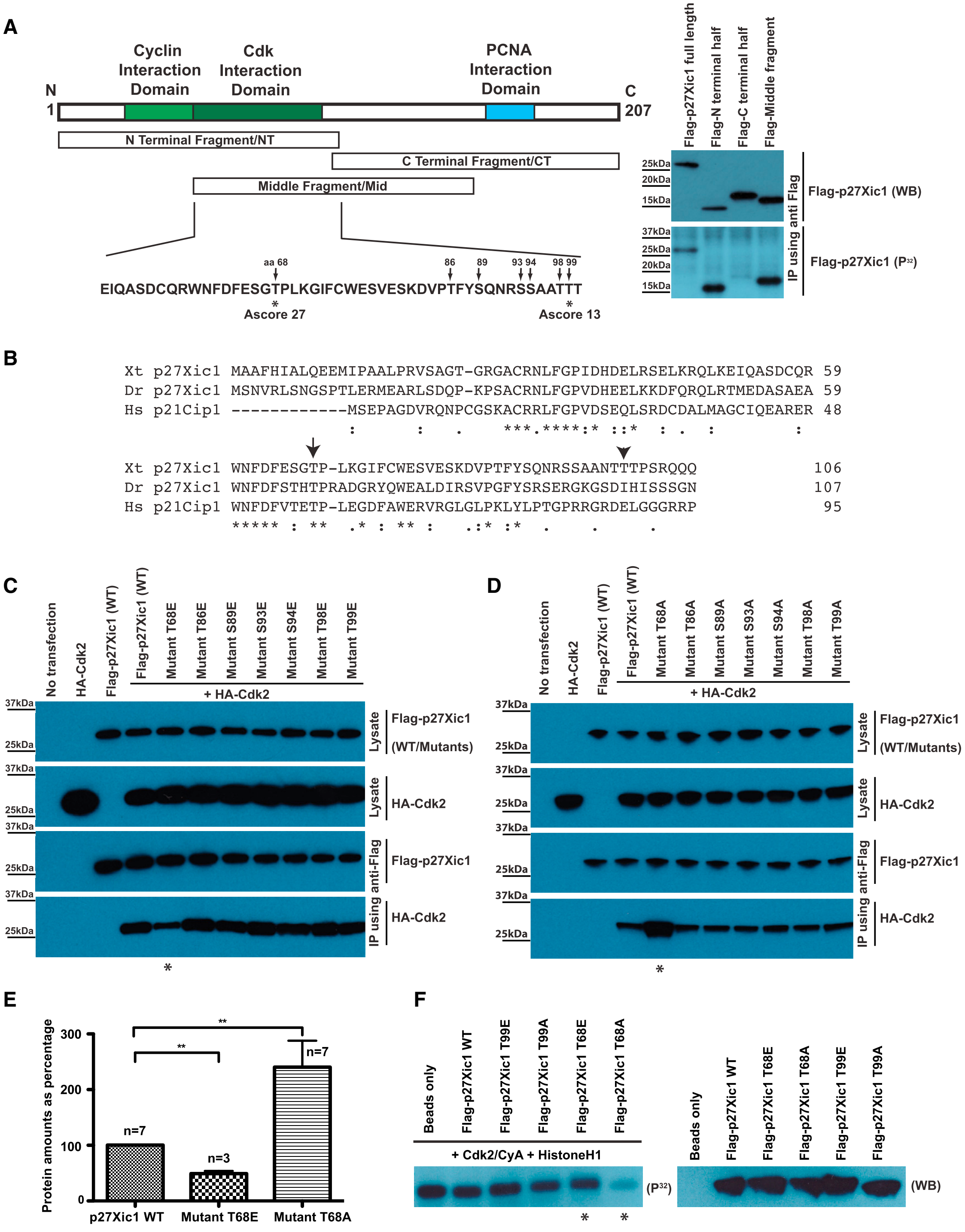

Figure 4. aPKC Phosphorylates p27Xic1 in Its Cdk-Interaction Domain, Leading to Its Weaker Binding and Inhibition of Cdk2

(A) The schematic shows p27Xic1 protein with its key interaction domains. Boxes in the middle show N-terminal, C-terminal and middle fragments that were used for immunocomplex kinase assays that indicated that aPKC phosphorylates p27Xic1 in its N-terminal half of the protein. The amino acid sequence underneath represents the Cdk-interaction domain of p27Xic1. MS analysis of Flag-p27Xic1 post-kinase assays identified multiple phosphorylation sites within its Cdk interaction domain with significant confidence scores (Ascores $\geq$ 13) after aPKC phosphorylation of p27Xic1 in vitro (indicated by arrows), whereas phosphosite (T68) was picked with Ascore of 27 after in vivo kinase assay; another phosphosite (T99) was picked with Ascore of 13 (both indicated by asterisks).

(B) Sequence alignment of the N-terminal half of *Xenopus* p27Xic1 with other CIP/KIP CdkIs shows that aPKC phosphosite T68 (with arrow) in p27Xic1 is conserved in mammalian p21Cip1 and fish p27Xic1; site T99 (with arrowhead) shows no conservation. Asterisks indicate fully conserved residues, whereas colons and periods indicate groups with strongly and weekly similar properties, respectively.

(legend continued on next page)

(Figure 4F). Taken together, these results mean that phosphorylation of p27Xic1 by aPKC essentially inhibits the activity of p27Xic1 by reducing its binding to Cdk2, which in turn results in failure to negatively regulate the Cdk2 kinase activity.

p27Xic1 Increases Neuronal Differentiation, and This Effect Is Rescued by aPKC

p27Xic1 overexpression by injecting mRNA has been shown to promote neurogenesis (Vernon et al., 2003), while its morpholino-mediated knockdown blocked neurogenesis and promoted progenitor proliferation (Carruthers et al., 2003). Here, we have used DNA injections, because in *Xenopus* the G1 phase appears in post-mid-blastula transition cell cycles. We found that such p27Xic1 DNA injections promoted neurogenesis (Figure 5A) in a cell autonomous fashion (Figure S6) and suppressed neural progenitor proliferation, consistent with results obtained previously with RNA overexpression.

A subset of p27Xic1-overexpressing embryos was used for fluorescence-activated cell sorting (FACS) cell-cycle profiling on isolated nuclei. Experimental embryos showed significantly higher percentage of nuclei in G1 phase than the control embryos (35.4% versus 27.4%), with a concomitant decrease of nuclei in S phase (40.7% versus 49.7% for control) and negligible effect on the proportion of nuclei in G2 phase (23.9% versus 22.9% for control, Figure 5B), indicating that G1 phase elongates and proliferation decreases upon p27Xic1 overexpression.

Whereas 89% of p27Xic1-injected embryos showed enhanced staining for *ElrC*, the percentage dropped to 33% with a milder phenotype when p27Xic1 was coinjected with aPKC-CAAX (Figure 5C). Thus, p27Xic1's overexpression phenotype of promoting neuronal differentiation was rescued by unilateral injection of aPKC-CAAX. The corresponding numbers for *N-tubulin* staining were 88% and 20% after overexpression of p27Xic1 alone or with aPKC-CAAX (data not shown). This supported the idea that aPKC promotes progenitor proliferation, at the expense of neuronal differentiation upstream of p27Xic1, by inhibiting its activity. The idea was further supported by the observation that the phosphomutant of p27Xic1 (T68A) could promote neuronal differentiation similar to p27Xic1, but aPKC-CAAX could not effectively rescue this effect (Figure 5C).

Lengthening the Cell Cycle via CDK Inhibition by Chemical Means Promotes Differentiation

To understand if the effects of aPKC and p27Xic1 activities on neuronal differentiation were mediated via their effects on the cell-cycle length, we manipulated the cell cycle of embryos by nongenetic, chemical means using Olomoucine (Olo), an inhibitor that lengthens the G1 phase by inhibiting the activity of G1 kinases (Calegari and Huttner, 2003). FACS analysis of nuclei from embryos treated with Olo showed that experimental embryos had more nuclei in G1 and G2 phases than control embryos, with a corresponding decrease in the proportion of nuclei in S phase, suggesting a decrease in proliferation and an elongation of growth phases (Figure 6A). Experimental embryos also showed a significantly higher number of MyT1-positive cells (a marker of differentiated neurons, 12.53 ± 2.44 per section) than the DMSO controls (8.75 ± 0.38) (Figures 6B and 6C), supporting the idea that elongating G1 phase promotes differentiation.

DISCUSSION

Cell polarization and cell division are two fundamental biological processes that have been independently linked to cellular differentiation. In this work, we showed that apicobasal polarity and cell-cycle control are directly linked in neural progenitor cells in a way that leads to distinct differentiation potential of polarized versus nonpolar progenitors. Our work has uncovered a remarkable similarity in the endogenous cell-cycle kinetics of apicobasally polarized and nonpolar progenitors between *Xenopus* neuroectoderm and the mouse embryonic cortex (Arai et al., 2011). In both cases, the total length of the cell cycle and the length of G1 in apicobasally polar progenitors (apical progenitors in the mouse, superficial progenitors in *Xenopus)* are shorter than those found in nonpolar ones (basal progenitors in the mouse, deep progenitors in *Xenopus*). In addition, in both cases, the basal nonpolar progenitors have a higher propensity to differentiate than the apicobasally polarized ones (Chalmers et al., 2002; Chenn and McConnell, 1995). Thus, although the *Xenopus* neuroectoderm shows a much simpler structure, some of the basic principles relating to the cell-cycle control during neurogenesis are highly conserved.

We have used this system to specifically address the role of the apicobasal polarity in controlling the cell cycle via the apically localized key kinase aPKC. aPKC is a ubiquitous kinase that gets activated in cell cortex in a PI-3,4,5-trisphosphate (PIP3)-dependent manner (reviewed by Hirai and Chida, 2003). Polarized cells are thought to contain a highly active pool of aPKC because it is recruited to the apical cortex and/or the junctional complexes (Joberty et al., 2000; Lin et al., 2000) where it should get activated. Therefore membrane targeting of aPKC by attaching it to a CAAX motif makes it constitutively active and mimics the effects of apicobasal polarization on aPKC activity (Lee et al., 2006; Sabherwal et al., 2009).

aPKC was the first molecule shown to promote the proliferation of polarized neuroblasts in *Drosophila* and neural progenitors in *Xenopus* both by gain- and loss-of-function experiments (Rolls et al., 2003; Chabu and Doe, 2008, 2009; Sabherwal et al., 2009). From a mechanistic point of view, the interaction of apicobasal polarity kinase aPKC with the cell-cycle machinery in polarized cells was thought to be indirect, via the Hippo pathway (reviewed in Genevet and Tapon, 2011). For example, aPKC phosphorylates and negatively regulates KIBRA, an upstream positive regulator of the Hippo pathway (Büther et al., 2004;

(C–E) In coIP assays performed in HeLa cells, phosphomimetic mutant for the site (T68E) showed reduced binding against Cdk2, whereas phosphomutant for the same site (T68A) showed enhanced binding against Cdk2. See also Figure S5. Quantitation for binding of wild-type and mutants p27Xic1 is shown in (E) (mean ± SEM).

(F) Histone H1 kinase assay shows that phosphomutant T68A of p27Xic1 strongly inhibits Cdk2's kinase activity, whereas phosphomimetic p27Xic1 (T68E) had negligible effect on the kinase activity of Cdk2, in comparison to the wild-type and T99 mutants of p27Xic1. Left blot shows the autoradiogram while the right blot shows the corresponding IP/western blot control. See also Figure S4.

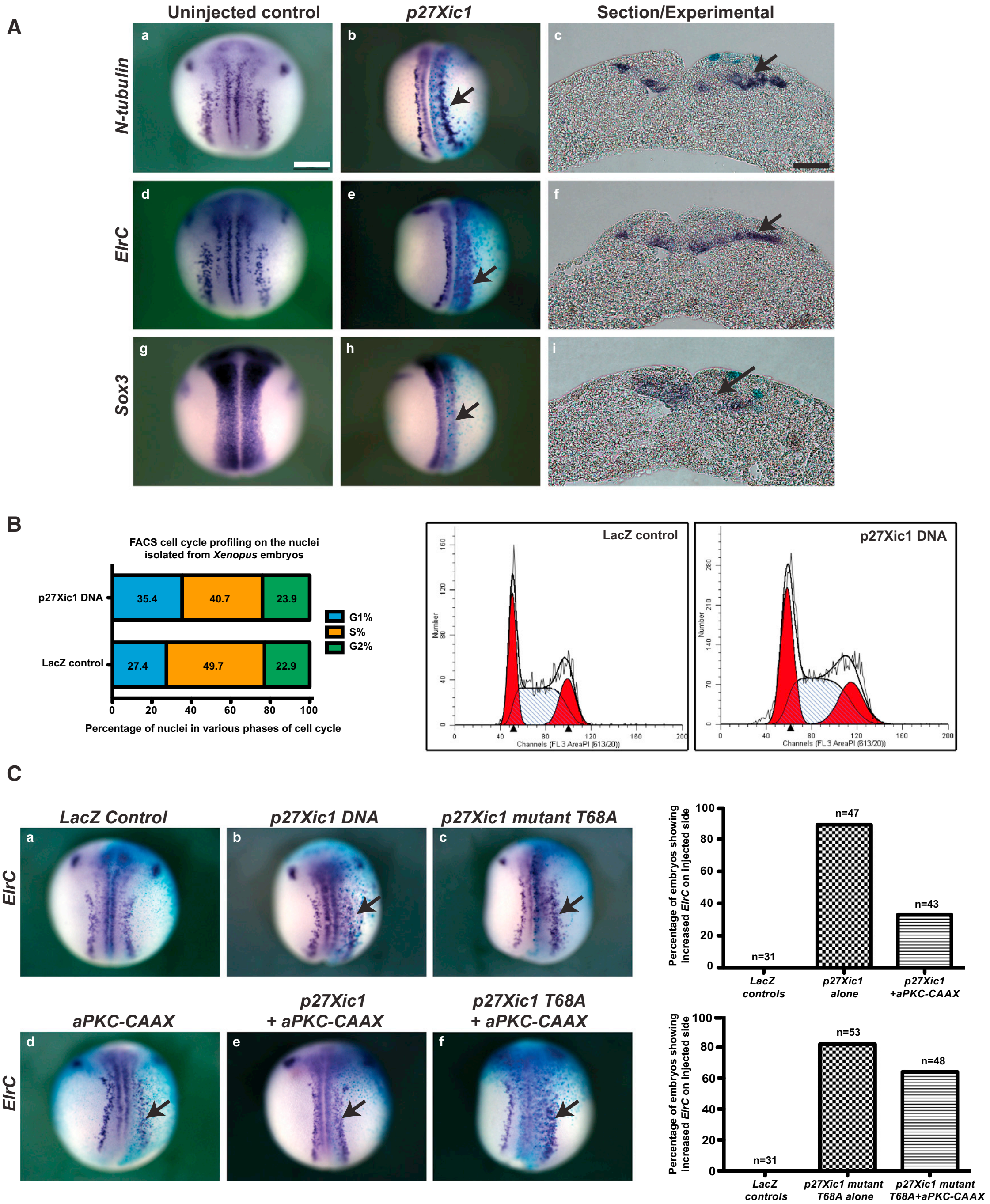

Figure 5. p27Xic1 Overexpression Promotes Neuronal Differentiation, and This Effect Is Rescued by Overexpression of aPKC-CAAX

(A) The neuronal markers *N-tubulin* and *ElrC* were increased and progenitor marker *Sox3* was decreased on the p27Xic1 injected (right; arrows) side in the embryos, as seen on whole mounts and sections. See also Figure S6.

(B) FACS cell-cycle profiling on p27Xic1-overexpressing embryos shows a significantly higher percentage of nuclei in the G1 phase, compared to control embryos, suggesting an elongation of G1.

(C) The increase in neuronal differentiation, observed by p27Xic1 overexpression, is rescued by coexpression of aPKC-CAAX, but it could not effectively rescue the ectopic neurogenesis caused by overexpression of phosphomutant p27Xic1 (T68A). Scale bars in (Aa) and (Ac) represent 500 μM and 50 μM respectively.

Yoshihama et al., 2011). Overexpression of aPKC mislocalizes apical Hippo to the cytoplasm with its negative regulator RASSF, leading to the dampening of the Hippo pathway and resulting in enhanced cell proliferation (Grzeschik et al., 2010).

Our current results establish a direct link between aPKC and the cell cycle in context of cell polarity. In this study, we show that aPKC-CAAX directly phosphorylates the nuclear cell cycle/cyclin-dependent kinase inhibitor p27Xic1. Phosphorylation

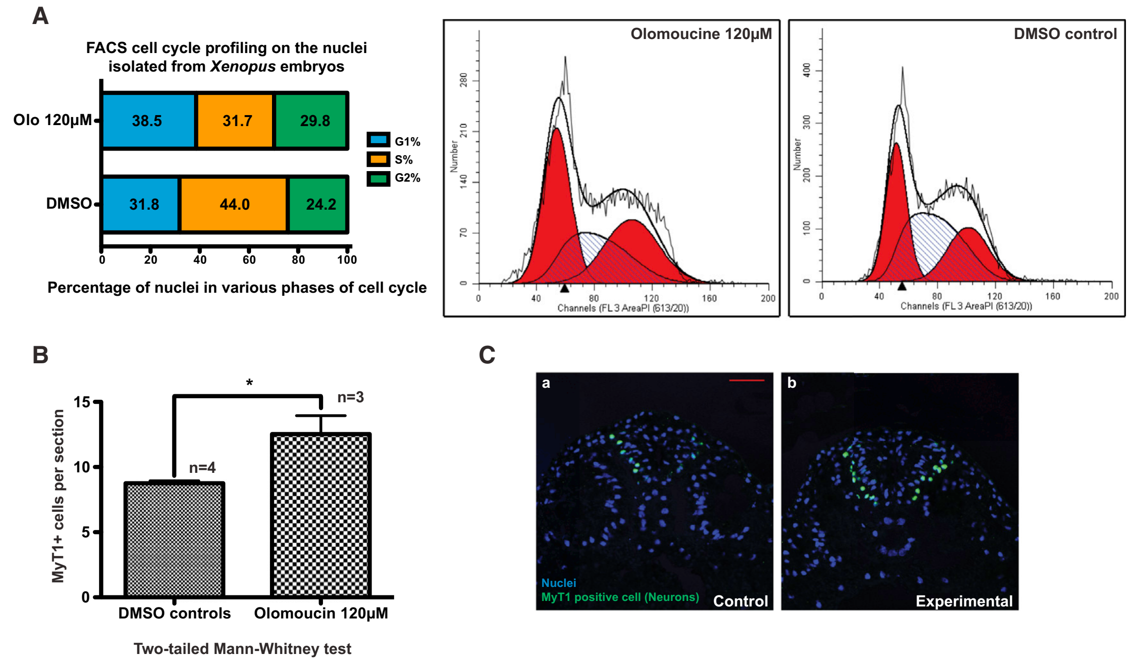

Figure 6. Elongating G1 Phase by Nongenetic Means Promotes Neuronal Differentiation

(A) Growing *Xenopus* embryos from NF10 to 20 in the presence of 120 µM olomoucine, an inhibitor of cyclin-dependent kinases and G1 progression, may elongate the G1 phase of the cell cycle, as indicated by the cell-cycle profiling of the nuclei isolated from NF20 embryos. Experimental embryos showed a higher number of nuclei in the G1 phase of the cell cycle than the control embryos.

(B) Experimental embryos, when stained with neuronal differentiation marker MyT1, showed a significantly higher number of differentiated neurons in comparison to control (mean ± SEM).

(C) Examples of sections from control and experimental embryos stained with MyT1 antibody (green). Nuclei are blue and scale bar represents 50 µM.

primarily takes place in T68, located in the N-terminal domain, although we cannot exclude contribution from other sites. aPKC-CAAX overexpression leads to a faster cell cycle in the neuroectoderm with shorter G1 and S phases, decreased neuronal differentiation, and enhanced neural proliferation (this work and Sabherwal et al., 2009).

aPKC has also been shown to have a role in cell proliferation of nonpolarized cells. For example, aPKC has been shown to directly phosphorylate and degrade p21Cip1 in human colorectal (HCT116) cells (Scott et al., 2002), to upregulate CyD1 transcription, and to reduce p27Kip1's nuclear translocation in a Ras-dependent manner in MCF7 cells (Castoria et al., 2004). We assume that these nonpolar cells have a basal activity of aPKC, which is further enhanced by recruitment to the apical membrane in polarized cells.

How does phosphorylation of p27Xic1 by polarity kinase aPKC lead to a faster cell cycle with shorter G1 and S phases? p27Xic1 physically interacts with Cdks and cyclins through its Cdk/Cy interaction domains and reduces the activities of G1 kinase (Cdk4/CyD), G1-S transition kinase (Cdk2/CyE) and S progression kinase (Cdk2/CyA) with different half-maximal inhibitory concentration values (Su et al., 1995; Finkielstein et al., 2001). Our data show that phosphomimetic p27Xic1 has a reduced ability to bind and inhibit Cdk2. Enhanced Cdk2 activity through CyE overexpression causes the cells to cycle faster through G1 and enter S phase prematurely (Ohtsubo and Roberts, 1993), whereas inhibition of CyE/Cdk2 complex delays entry into S phase (van den Heuvel and Harlow, 1993). Thus, inhibition of Cdk2 kinase activity by CdkIs, like p27Xic1, is key in inhibiting the cycling of progenitors and promoting their differentiation. We suggest that in polarized progenitors, unrestricted kinase activities of G1/S-specific Cdk/Cy complexes due to lower p27Xic1 activity would shorten their G1 phase and concomitantly promote S phase entry, thereby promoting their cycling and interfering with the ability to differentiate.

The prolongation of total cell-cycle length, and G1 in particular, as cells approach differentiation seems to be a widespread phenomenon (Takahashi et al., 1995; Coronado et al., 2013; Roccio et al., 2013). Mechanistically, it is thought that elongation of the G1 phase promotes neuronal differentiation by providing sufficient time for the accumulation and posttranscriptional modification of differentiation-promoting factors, such as Neurogenin and NeuroD (reviewed in Lange and Calegari, 2010; Hardwick and Philpott, 2014). In support of this idea, it was recently shown that the activity of Neurogenin is controlled by time-dependent, rheostat-like sequential (de)phosphorylation events in a cell-cycle-dependent manner (Ali et al., 2011).

In conclusion, our findings provide mechanistic evidence for a direct link between apicobasal polarity and the cell cycle through aPKC and p27Xic1, which is used during development to endow polarized neural progenitors with lower propensity for differentiation than their nonpolar counterparts. p27Xic1 has a complex mode of regulation, which includes phosphorylation, ubiquitination, and degradation in the nucleus, and changes in the activity

through the cell cycle (Chuang and Yew, 2001; Chuang et al., 2005; Lin et al., 2006). Regulation of aPKC is likely to be equally complex, including shuttling between the membrane and the nucleus (Sabherwal et al., 2009). One challenge for the future is to understand the dynamic interaction between aPKC and p27Xic1 during the cell cycle.

EXPERIMENTAL PROCEDURES

Cell-Cycle Length Analysis

Dual-Pulse S Phase Labeling

This method enables the estimation of the total cell-cycle length (T_C) and S phase length (T_S) by injecting two different thymidine analogs (Martynoga et al., 2005). Embryos collected at open neural plate stage (NF13) were injected with ethynyl deoxyuridine (EdU, 2 × 10 nl, 10 mM in DMSO [Life Technologies]) in the neural plate midline at two different positions. After 2 hr incubation, bromodeoxyuridine (BrdU, 2 × 10 nL, 10 mM in DMSO [Roche]) was injected in the same place (Figure 1A). Embryos were fixed in MEMFA 30 min later and processed for EdU, BrdU, and Sox3 (as a marker of both polar and nonpolar neural progenitors) staining as described elsewhere (Auger et al., 2012).

Percentage of Labeled Mitoses Analysis

This method involves labeling the mitotic (phosphohistone H3/PH3 +) cells with a thymidine analog (EdU or BrdU) and gives an estimate of the lengths of total cell cycle, S and G2+1/2M phases, depending on the duration of the experiment (Shackney and Ritch, 1987; Peco et al., 2012). Embryos at NF13 were injected with EdU as described above and collected every 30 min until 240 min as the last point of our experiment (Figure 1B). Embryos were fixed, sectioned, and stained for EdU, PH3, and Sox3 as described elsewhere (Auger et al., 2012).

Cell-Cycle Profiling using Fluorescence-Activated Cell Sorting Analysis on Propidium Iodide-Stained Nuclei

Nuclei from NF15 embryos (20 per set) for FACS analysis were prepared as described elsewhere (Frederick and Andrews, 1994), followed by cell-cycle profiling using a CyAn ADP flow cytometer from Beckman Coulter running Summit v4.3 software. The modfit analysis on the data was carried out using FloJo software.

Live-Cell Imaging of HeLa Cells-Based Fucci Biosensor for Cell-Cycle Length Analysis

HeLa Fucci cells were maintained and propagated as described elsewhere (Sakaue-Sawano et al., 2008). Control and experimental cells with inhibitors were imaged on a heated stage at 37°C and 5% CO_2 supply using NikonA1 confocal microscope for 2–3 days.

Imaging and Data Analysis

For dual-pulse S phase labeling and percentage of labeled mitoses analyses, five to ten sections per embryo from the mid-anterior neural plate were imaged on a NikonA1 confocal microscope. Still and time-lapse (for Fucci) images were processed and analyzed using ImageJ software. All the statistical tests were performed using Prism graph-pad software. Normality of the data was checked using D'Agostino & Pearson omnibus normality test. Depending on the outcome of the normality test, appropriate tests for significance (mentioned in the figure legends) were chosen from the software and applied to the data.

Overexpression in Whole Embryos, X-Gal Staining, and Whole Mount In Situ Hybridization Followed by Sectioning

Animal experiments were approved by the University's Ethical Review Panel and were undertaken under UK Home Office project license PPL 70/7648. Culturing and overexpression in *Xenopus laevis* embryos were carried out as described elsewhere (Sabherwal et al., 2009). Staging was made according to the Nieuwkoop and Faber table of development (NF stages; Nieuwkoop, 1967). The following amounts of mRNA or DNA were injected: *aPKC-CAAX* 0.25–0.5 ng, *NLS-aPKC-NT* 0.25–0.5 ng, and *p27Xic1* 0.15 ng (0.075ng × 2); 0.5 ng *GFP* or *β-galactosidase (LacZ)* mRNA was coinjected as lineage tracer/control. Antisense probes *N-tubulin*, *ElrC*, *and Sox3* have been described (Chalmers et al., 2002; Carruthers et al., 2003). Fixation, X-gal staining, in situ hybridization, and sectioning were carried out as described elsewhere (Bourguignon et al., 1998).

Expression and Purification of GST-Tagged p27Xic1, In Vitro and In Vivo Kinase Assays

GST-p27Xic1 was expressed in BL21 *Escherichia coli* cells and purified as described by the plasmid supplier (GE Healthcare). For in vitro kinase assay, 20 μl of eluted GST-p27Xic1 was mixed with kinase buffer (20 mM Tris-HCl [pH 7.5] and 5 mM $MgCl_2$), GST-aPKC (1 μl/0.1 μg, Calbiochem)/His-aPKC (1 μl/0.1 μg, Millipore), and γP32-radiolabelled ATP (1 μl, 0.37 MBq [Perkin Elmer]). The final 30 μl reaction was incubated at 30°C for 30 min, boiled with sample buffer, and loaded on 10%–12% polyacrylamide gel. Post-run, dried gel on Whatman paper was used to expose X-ray film to get an autoradiogram impression of the kinase reaction.

To perform in vitro kinase assays in an embryonic environment, the reaction was performed exactly as described above, except that instead of kinase buffer, NF13-14 embryonic lysate (as described below but without EDTA and EGTA) was used as the incubation medium with 10 μl (3.7 MBq) of γP32-radiolabelled ATP.

For in vivo kinase assays, HeLa cells were transfected with Flag-p27Xic1 alone or with HA-aPKC-CAAX. For inhibitor treatment, Flag-p27Xic1 transfected cells were incubated with the media containing the inhibitor for 24 hr. γP32-radiolabelled orthophosphate (Perkin Elmer) incorporation was performed as described elsewhere (Barilá et al., 2000), followed by immunoprecipitation of Flag-p27Xic1 and autoradiography as described above.

For the immunocomplex kinase assay, 10 μl of protein A/G Sepharose beads (Santa Cruz Biotechnology) was mixed with 100 μl of cleared lysate from HeLa cells (overexpressing the protein of interest) and 2–5 μg of mouse anti-Flag antibody (M2 clone [Sigma]). The tube was rotated overnight at 4°C. Washed beads were incubated with the kinase reaction mix as described above. Gel was run, dried, and exposed as described above.

For Histone H1 in vitro kinase assay, 0.5 μg of Histone H1 (Sigma), 100 ng of active Cdk2/CyclinA protein (Millipore), and 1 μl of γP32-radiolabelled ATP (0.37 MBq) were mixed with immunoprecipitated wild-type or mutant p27Xic1 in 1× kinase buffer and incubated at 30°C for 30 min. The reaction was stopped and autoradiography was performed as described above.

Cell Culture, Transfections, Coimmunoprecipitation, Whole Embryo Lysates, and Western Blot Analysis

HeLa cells were maintained and transfected as described elsewhere (Sabherwal et al., 2009). CoIP assays from HeLa cells overexpressing proteins of interest were performed as described elsewhere (Roth et al., 2010). Proteins were immunoprecipitated using 5–10 μg of the following antibodies: mouse anti-Flag (M2 clone [Sigma]), mouse anti-HA (Sigma), and mouse anti-Myc (Santa Cruz Biotechnology). CoIP from embryos was performed using the Pierce Crosslink IP kit, following the manufacturer's protocol using rabbit anti-aPKC (C20 clone, Santa Cruz Biotechnology) and rabbit anti-p27Xic1 antibodies (custom made against the antigen as described by Shou and Dunphy, 1996).

For making embryo lysates, embryos were dissociated in lysis buffer (50 mM Tris pH7.5 + 150 mM NaCl + 0.5% NP40 + 5 mM EDTA + 5 mM EGTA). Cleared lysates were used for western blot analysis. The following antibodies were used for detection: rat HA-HRP (Roche), mouse Flag-HRP (Sigma), mouse Myc-HRP (Santa Cruz Biotechnology), rabbit aPKC (C20 clone, Santa Cruz Biotechnology), rabbit anti-p27Xic1, and mouse anti-α-tubulin (DM1A clone [Sigma]).

Chemical Inhibitors

Myristoylated pseudosubstrate inhibitor against aPKC (Invitrogen Life Technologies; Sajan et al., 1999) was used at a working concentration of 12.5 μM. Chemical inhibitor against aPKC (Gö6983 [Calbiochem] Saiz et al., 2013) was used at a working concentration of 5 μM. Olomoucine (Calbiochem; Calegari and Huttner, 2003) was used at a working concentration of 120 μM.

Whole-Mount Antibody Staining, Cryosectioning, and Antibody Staining on Sections

Antibody staining on sections and whole mounts was performed as described elsewhere (Sabherwal et al., 2009). The following primary and secondary antibodies were used: rat anti-HA (Roche), rabbit anti-Flag (Sigma), mouse anti-BrdU (MoBu clone [Life Technologies]), mouse anti-phosphohistoneH3 (Abcam) and rabbit anti-Sox3 (custom made; Zhang et al., 2003), rabbit

anti-MyT1 (custom made; Sabherwal et al., 2009), Alexa488-coupled anti-mouse, and Alexa647-coupled anti-rabbit (Life Technologies).

Mass Spectroscopic Analysis

For phosphopeptide mapping, protein bands were Coomassie stained, excised and in situ digested with trypsin. LC/MS/MS analysis of phosphosites was performed at the Taplin Biological Mass Spectrometry Facility (Harvard Medical School) as described (Roig et al., 2005). Please refer to the Supplemental Experimental Procedures for information regarding sample preparation for MS analysis.

SUPPLEMENTAL INFORMATION

Supplemental Information includes Supplemental Experimental Procedures, six figures, and one movie and can be found with this article online at http://dx.doi.org/10.1016/j.devcel.2014.10.023.

ACKNOWLEDGMENTS

We thank Atsushi Miyawaki (Brain Science Institute, RIKEN, Saitama, Japan) for kindly gifting us the HeLa Fucci biosensor. This work was funded by a Wellcome Trust Senior Fellowship grant (WT090868 to N.P.). N.S. and R.T are Wellcome Trust research associates and R.L. and P.S. are research assistants with the N.P. lab.

Received: September 16, 2013
Revised: September 5, 2014
Accepted: October 29, 2014
Published: December 8, 2014

REFERENCES

Alberts, B., Johnson, A., Lewis, J., Raff, M., Roberts, K., and Walter, P. (2002). Molecular Biology of the Cell. (New York: Garland Science).

Ali, F., Hindley, C., McDowell, G., Deibler, R., Jones, A., Kirschner, M., Guillemot, F., and Philpott, A. (2011). Cell cycle-regulated multi-site phosphorylation of Neurogenin 2 coordinates cell cycling with differentiation during neurogenesis. Development *138*, 4267–4277.

Arai, Y., Pulvers, J.N., Haffner, C., Schilling, B., Nüsslein, I., Calegari, F., and Huttner, W.B. (2011). Neural stem and progenitor cells shorten S-phase on commitment to neuron production. Nat. Commun. *2*, 154.

Auger, H., Thuret, R., Yakoubi, W.E., and Papalopulu, N. (2012). A Bromodeoxyuridine (BrdU) based protocol for characterizing proliferating progenitors in *Xenopus* embryos. In Methods in Molecular Biology (Totowa: Humana Press), pp. 461–475.

Barilá, D., Mangano, R., Gonfloni, S., Kretzschmar, J., Moro, M., Bohmann, D., and Superti-Furga, G. (2000). A nuclear tyrosine phosphorylation circuit: c-Jun as an activator and substrate of c-Abl and JNK. EMBO J. *19*, 273–281.

Beausoleil, S.A., Villén, J., Gerber, S.A., Rush, J., and Gygi, S.P. (2006). A probability-based approach for high-throughput protein phosphorylation analysis and site localization. Nat. Biotechnol. *24*, 1285–1292.

Bourguignon, C., Li, J., and Papalopulu, N. (1998). XBF-1, a winged helix transcription factor with dual activity, has a role in positioning neurogenesis in Xenopus competent ectoderm. Development *125*, 4889–4900.

Büther, K., Plaas, C., Barnekow, A., and Kremerskothen, J. (2004). KIBRA is a novel substrate for protein kinase Czeta. Biochem. Biophys. Res. Commun. *317*, 703–707.

Calegari, F., and Huttner, W.B. (2003). An inhibition of cyclin-dependent kinases that lengthens, but does not arrest, neuroepithelial cell cycle induces premature neurogenesis. J. Cell Sci. *116*, 4947–4955.

Carruthers, S., Mason, J., and Papalopulu, N. (2003). Depletion of the cell-cycle inhibitor p27(Xic1) impairs neuronal differentiation and increases the number of ElrC(+) progenitor cells in Xenopus tropicalis. Mech. Dev. *120*, 607–616.

Castoria, G., Migliaccio, A., Di Domenico, M., Lombardi, M., de Falco, A., Varricchio, L., Bilancio, A., Barone, M.V., and Auricchio, F. (2004). Role of atypical protein kinase C in estradiol-triggered G1/S progression of MCF-7 cells. Mol. Cell. Biol. *24*, 7643–7653.

Chabu, C., and Doe, C.Q. (2008). Dap160/intersectin binds and activates aPKC to regulate cell polarity and cell cycle progression. Development *135*, 2739–2746.

Chabu, C., and Doe, C.Q. (2009). Twins/PP2A regulates aPKC to control neuroblast cell polarity and self-renewal. Dev. Biol. *330*, 399–405.

Chalmers, A.D., Welchman, D., and Papalopulu, N. (2002). Intrinsic differences between the superficial and deep layers of the Xenopus ectoderm control primary neuronal differentiation. Dev. Cell *2*, 171–182.

Chenn, A., and McConnell, S.K. (1995). Cleavage orientation and the asymmetric inheritance of Notch1 immunoreactivity in mammalian neurogenesis. Cell *82*, 631–641.

Chuang, L.C., and Yew, P.R. (2001). Regulation of nuclear transport and degradation of the Xenopus cyclin-dependent kinase inhibitor, p27Xic1. J. Biol. Chem. *276*, 1610–1617.

Chuang, L.-C., Zhu, X.-N., Herrera, C.R., Tseng, H.-M., Pfleger, C.M., Block, K., and Yew, P.R. (2005). The C-terminal domain of the Xenopus cyclin-dependent kinase inhibitor, p27Xic1, is both necessary and sufficient for phosphorylation-independent proteolysis. J. Biol. Chem. *280*, 35290–35298.

Coronado, D., Godet, M., Bourillot, P.-Y., Tapponnier, Y., Bernat, A., Petit, M., Afanassieff, M., Markossian, S., Malashicheva, A., Iacone, R., et al. (2013). A short G1 phase is an intrinsic determinant of naïve embryonic stem cell pluripotency. Stem Cell Res. (Amst.) *10*, 118–131.

Dutta, D., Ray, S., Home, P., Larson, M., Wolfe, M.W., and Paul, S. (2011). Self-renewal versus lineage commitment of embryonic stem cells: protein kinase C signaling shifts the balance. Stem Cells *29*, 618–628.

Eckert, J.J., McCallum, A., Mears, A., Rumsby, M.G., Cameron, I.T., and Fleming, T.P. (2004). PKC signalling regulates tight junction membrane assembly in the pre-implantation mouse embryo. Reproduction *127*, 653–667.

Finkielstein, C.V., Lewellyn, A.L., and Maller, J.L. (2001). The midblastula transition in Xenopus embryos activates multiple pathways to prevent apoptosis in response to DNA damage. Proc. Natl. Acad. Sci. USA *98*, 1006–1011.

Folch, J., Junyent, F., Verdaguer, E., Auladell, C., Pizarro, J.G., Beas-Zarate, C., Pallàs, M., and Camins, A. (2012). Role of cell cycle re-entry in neurons: a common apoptotic mechanism of neuronal cell death. Neurotox. Res. *22*, 195–207.

Frederick, D.L., and Andrews, M.T. (1994). Cell cycle remodeling requires cell-cell interactions in developing Xenopus embryos. J. Exp. Zool. *270*, 410–416.

Genevet, A., and Tapon, N. (2011). The Hippo pathway and apico-basal cell polarity. Biochem. J. *436*, 213–224.

Grzeschik, N.A., Parsons, L.M., Allott, M.L., Harvey, K.F., and Richardson, H.E. (2010). Lgl, aPKC, and Crumbs regulate the Salvador/Warts/Hippo pathway through two distinct mechanisms. Curr. Biol. *20*, 573–581.

Hardwick, L.J.A., and Philpott, A. (2014). Nervous decision-making: to divide or differentiate. Trends Genet. *30*, 254–261.

Hasan, S.M.M., Sheen, A.D., Power, A.M., Langevin, L.M., Xiong, J., Furlong, M., Day, K., Schuurmans, C., Opferman, J.T., and Vanderluit, J.L. (2013). Mcl1 regulates the terminal mitosis of neural precursor cells in the mammalian brain through p27Kip1. Development *140*, 3118–3127.

Hindley, C., and Philpott, A. (2012). Co-ordination of cell cycle and differentiation in the developing nervous system. Biochem. J. *444*, 375–382.

Hirai, T., and Chida, K. (2003). Protein kinase Czeta (PKCzeta): activation mechanisms and cellular functions. J. Biochem. *133*, 1–7.

Joberty, G., Petersen, C., Gao, L., and Macara, I.G. (2000). The cell-polarity protein Par6 links Par3 and atypical protein kinase C to Cdc42. Nat. Cell Biol. *2*, 531–539.

Kranenburg, O., Scharnhorst, V., Van der Eb, A.J., and Zantema, A. (1995). Inhibition of cyclin-dependent kinase activity triggers neuronal differentiation of mouse neuroblastoma cells. J. Cell Biol. *131*, 227–234.

Lange, C., and Calegari, F. (2010). Cdks and cyclins link G1 length and differentiation of embryonic, neural and hematopoietic stem cells. Cell Cycle *9*, 1893–1900.

Lange, C., Huttner, W.B., and Calegari, F. (2009). Cdk4/cyclinD1 overexpression in neural stem cells shortens G1, delays neurogenesis, and promotes the generation and expansion of basal progenitors. Cell Stem Cell *5*, 320–331.

Lee, C.Y., Robinson, K.J., and Doe, C.Q. (2006). Lgl, Pins and aPKC regulate neuroblast self-renewal versus differentiation. Nature *439*, 594–598.

Lin, D., Edwards, A.S., Fawcett, J.P., Mbamalu, G., Scott, J.D., and Pawson, T. (2000). A mammalian PAR-3-PAR-6 complex implicated in Cdc42/Rac1 and aPKC signalling and cell polarity. Nat. Cell Biol. *2*, 540–547.

Lin, H.R., Chuang, L.C., Boix-Perales, H., Philpott, A., and Yew, P.R. (2006). Ubiquitination of cyclin-dependent kinase inhibitor, Xic1, is mediated by the Xenopus F-box protein xSkp2. Cell Cycle *5*, 304–314.

Lobjois, V., Bel-Vialar, S., Trousse, F., and Pituello, F. (2008). Forcing neural progenitor cells to cycle is insufficient to alter cell-fate decision and timing of neuronal differentiation in the spinal cord. Neural Dev. *3*, 4.

Mairet-Coello, G., Tury, A., Van Buskirk, E., Robinson, K., Genestine, M., and DiCicco-Bloom, E. (2012). p57(KIP2) regulates radial glia and intermediate precursor cell cycle dynamics and lower layer neurogenesis in developing cerebral cortex. Development *139*, 475–487.

Martynoga, B., Morrison, H., Price, D.J., and Mason, J.O. (2005). Foxg1 is required for specification of ventral telencephalon and region-specific regulation of dorsal telencephalic precursor proliferation and apoptosis. Dev. Biol. *283*, 113–127.

Nieuwkoop, P.D. (1967). Normal Table of *Xenopus Laevis* (Daudin). (New York: Garland Pub).

Ohnuma, S., and Harris, W.A. (2003). Neurogenesis and the cell cycle. Neuron *40*, 199–208.

Ohtsubo, M., and Roberts, J.M. (1993). Cyclin-dependent regulation of G1 in mammalian fibroblasts. Science *259*, 1908–1912.

Peco, E., Escude, T., Agius, E., Sabado, V., Medevielle, F., Ducommun, B., and Pituello, F. (2012). The CDC25B phosphatase shortens the G2 phase of neural progenitors and promotes efficient neuron production. Development *139*, 1095–1104.

Roccio, M., Schmitter, D., Knobloch, M., Okawa, Y., Sage, D., and Lutolf, M.P. (2013). Predicting stem cell fate changes by differential cell cycle progression patterns. Development *140*, 459–470.

Roig, J., Groen, A., Caldwell, J., and Avruch, J. (2005). Active Nercc1 protein kinase concentrates at centrosomes early in mitosis and is necessary for proper spindle assembly. Mol. Biol. Cell *16*, 4827–4840.

Rolls, M.M., Albertson, R., Shih, H.P., Lee, C.Y., and Doe, C.Q. (2003). Drosophila aPKC regulates cell polarity and cell proliferation in neuroblasts and epithelia. J. Cell Biol. *163*, 1089–1098.

Roth, M., Bonev, B., Lindsay, J., Lea, R., Panagiotaki, N., Houart, C., and Papalopulu, N. (2010). FoxG1 and TLE2 act cooperatively to regulate ventral telencephalon formation. Development *137*, 1553–1562.

Sabherwal, N., and Papalopulu, N. (2012). Apicobasal polarity and cell proliferation during development. Essays Biochem. *53*, 95–109.

Sabherwal, N., Tsutsui, A., Hodge, S., Wei, J., Chalmers, A.D., and Papalopulu, N. (2009). The apicobasal polarity kinase aPKC functions as a nuclear determinant and regulates cell proliferation and fate during Xenopus primary neurogenesis. Development *136*, 2767–2777.

Saiz, N., Grabarek, J.B., Sabherwal, N., Papalopulu, N., and Plusa, B. (2013). Atypical protein kinase C couples cell sorting with primitive endoderm maturation in the mouse blastocyst. Development *140*, 4311–4322.

Sajan, M.P., Standaert, M.L., Bandyopadhyay, G., Quon, M.J., Burke, T.R., Jr., and Farese, R.V. (1999). Protein kinase C-zeta and phosphoinositide-dependent protein kinase-1 are required for insulin-induced activation of ERK in rat adipocytes. J. Biol. Chem. *274*, 30495–30500.

Sakaue-Sawano, A., Kurokawa, H., Morimura, T., Hanyu, A., Hama, H., Osawa, H., Kashiwagi, S., Fukami, K., Miyata, T., Miyoshi, H., et al. (2008). Visualizing spatiotemporal dynamics of multicellular cell-cycle progression. Cell *132*, 487–498.

Scott, M.T., Ingram, A., and Ball, K.L. (2002). PDK1-dependent activation of atypical PKC leads to degradation of the p21 tumour modifier protein. EMBO J. *21*, 6771–6780.

Shackney, S.E., and Ritch, P.S. (1987). Percent labeled mitosis curve analysis. In Techniques in Cell Cycle Analysis (New York: Springer), pp. 31–45.

Shou, W., and Dunphy, W.G. (1996). Cell cycle control by Xenopus p28Kix1, a developmentally regulated inhibitor of cyclin-dependent kinases. Mol. Biol. Cell *7*, 457–469.

Standaert, M.L., Bandyopadhyay, G., Sajan, M.P., Cong, L., Quon, M.J., and Farese, R.V. (1999). Okadaic acid activates atypical protein kinase C (ζ/λ) in rat and 3T3/L1 adipocytes. An apparent requirement for activation of Glut4 translocation and glucose transport. J. Biol. Chem. *274*, 14074–14078.

Su, J.Y., Rempel, R.E., Erikson, E., and Maller, J.L. (1995). Cloning and characterization of the Xenopus cyclin-dependent kinase inhibitor p27XIC1. Proc. Natl. Acad. Sci. USA *92*, 10187–10191.

Takahashi, T., Nowakowski, R.S., and Caviness, V.S.J., Jr. (1995). The cell cycle of the pseudostratified ventricular epithelium of the embryonic murine cerebral wall. J. Neurosci. *15*, 6046–6057.

van den Heuvel, S., and Harlow, E. (1993). Distinct roles for cyclin-dependent kinases in cell cycle control. Science *262*, 2050–2054.

Vernon, A.E., Devine, C., and Philpott, A. (2003). The cdk inhibitor p27Xic1 is required for differentiation of primary neurones in Xenopus. Development *130*, 85–92.

Wilcock, A.C., Swedlow, J.R., and Storey, K.G. (2007). Mitotic spindle orientation distinguishes stem cell and terminal modes of neuron production in the early spinal cord. Development *134*, 1943–1954.

Yoshihama, Y., Sasaki, K., Horikoshi, Y., Suzuki, A., Ohtsuka, T., Hakuno, F., Takahashi, S., Ohno, S., and Chida, K. (2011). KIBRA suppresses apical exocytosis through inhibition of aPKC kinase activity in epithelial cells. Curr. Biol. *21*, 705–711.

Zhang, C., Basta, T., Jensen, E.D., and Klymkowsky, M.W. (2003). The β-catenin/VegT-regulated early zygotic gene Xnr5 is a direct target of SOX3 regulation. Development *130*, 5609–5624.

Developmental Cell, Volume *31*

Supplemental Information

aPKC Phosphorylates p27Xic1, Providing a Mechanistic Link between Apicobasal Polarity and Cell-Cycle Control

Nitin Sabherwal, Raphael Thuret, Robert Lea, Peter Stanley, and Nancy Papalopulu

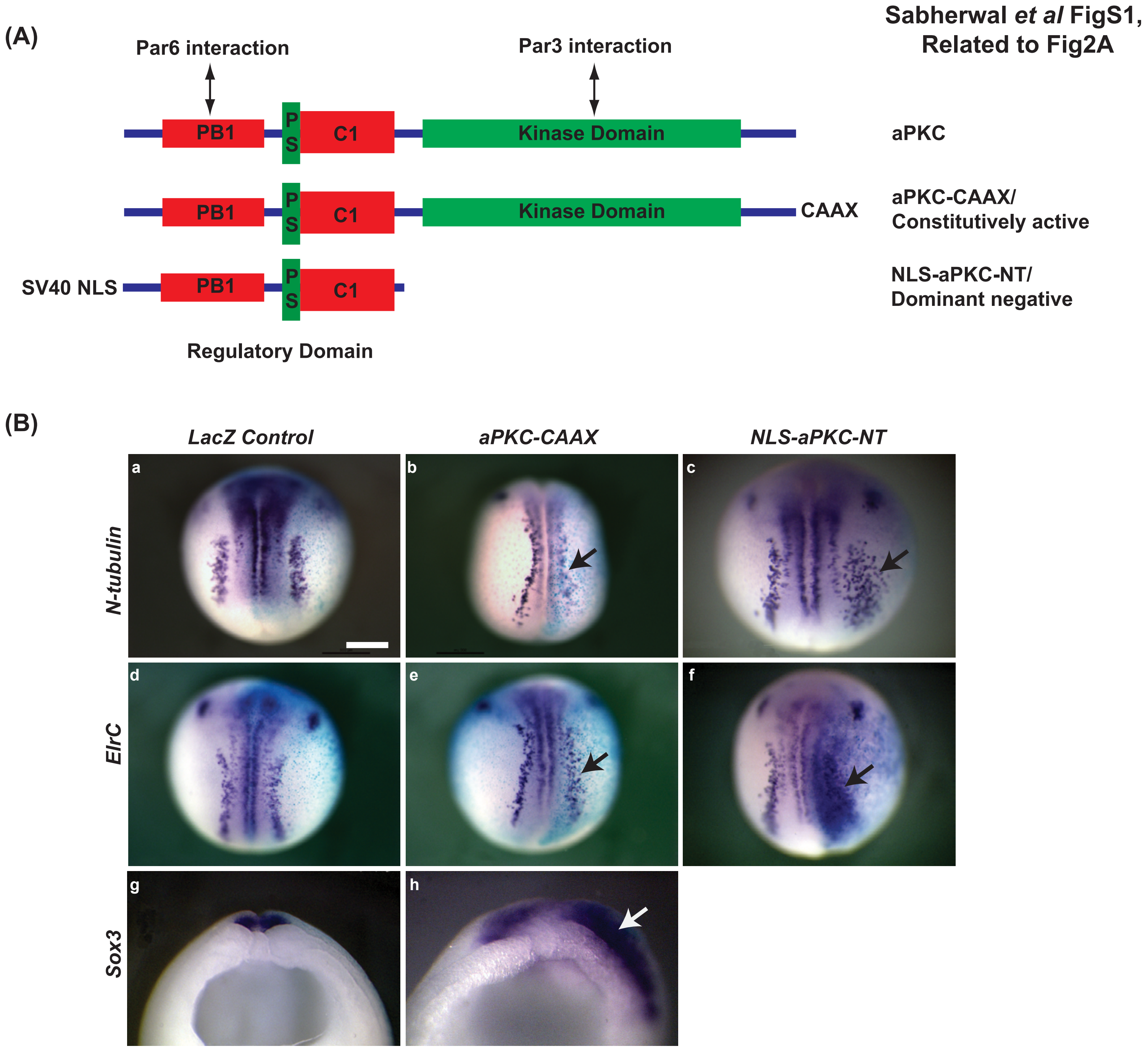

FigS1, related to fig2A: Over-expression of apicobasal polarity kinase aPKC promotes progenitor proliferation and suppresses neuronal differentiation. **(A)** shows wild type , constitutively active and dominant negative versions of aPKC with their important domains. PB1 domain (responsible for aPKC's interaction with Par6), PS/pseudosubstrate domain and C1 domain (responsible for its interaction with membrane lipids) constitute aPKC's N-terminal regulatory domain, while C terminal part of the protein constitutes its kinase domain. The schematic has been adapted from Suzuki et al, 2003, J. Biochem. 133, 9–16. Constitutively active and dominant negative forms of aPKC have been reported in our previous publication (Sabherwal *et al*, 2009). Overexpression of membrane targeted active aPKC (*aPKC-CAAX*) suppresses neuronal differentiation as differentiation markers *N-tubulin* and *ElrC* showed reduction on injected side of the embryos **(Bb and Be)**, and promoted neural progenitor proliferation (Progenitor marker *Sox3* was enhanced on injected side, **Bh**). Overexpressing nuclear dominant negative form of aPKC had the inverse effect of promoting differentiation **(Bc and Bf)**. Scale bar in Ba represents 500μM.

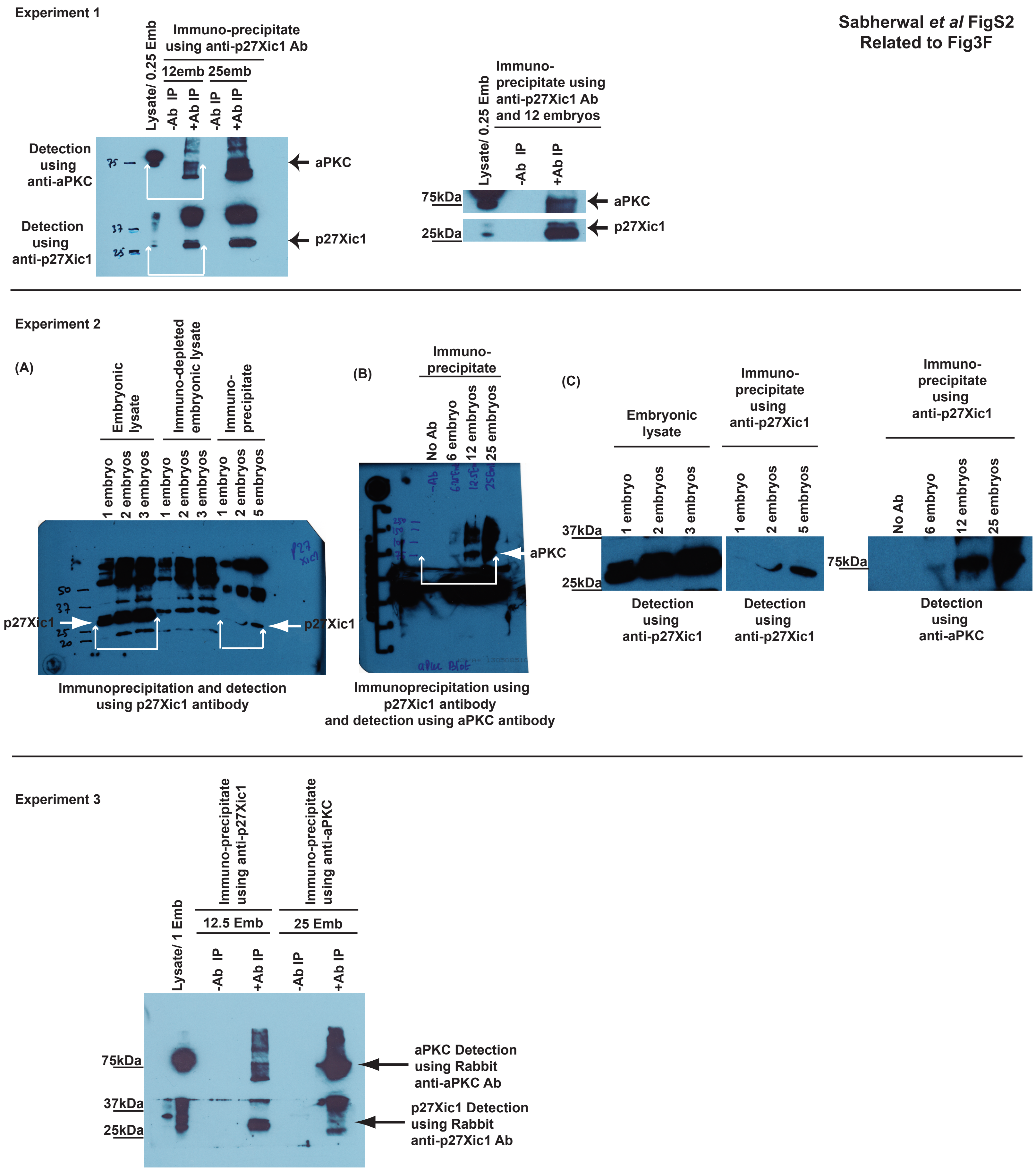

FigS2, related to fig3F: Physical interaction between p27Xic1 and aPKC was confirmed for endogenous proteins from embryonic lysates by a co-immuno-precipitation assay using antibodies against endogenous p27Xic1 and aPKC. In the first two experiments and last experiment (lanes 2 and 3), p27Xic1 was immuno-precipitated from embryonic lysate usinga home-made antibody against p27Xic1 and aPKC bound to p27Xic1 was detected using a commercially available aPKC antibody. Original blots for first two experiments are shown on the left hand side and the final blots are shown on right hand side. Final blot from the experiment 1 has been shown in fig3F. The last two lanes in experiment 3 show the inverse experiment where aPKC was immunoprecipitated using antibody against aPKC and p27Xic1 bound to it was detected using home-made antibody against p27Xic1. .

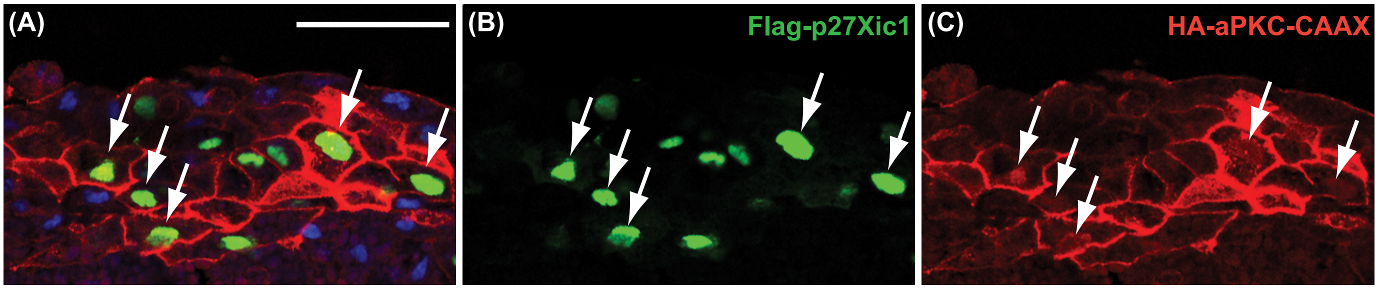

FigS3, related to fig3: Regulation of p27Xic1 activity by aPKC likely takes place in the cell nucleus. To see where in the cell p27Xic1 and aPKC-CAAX interact with each other, we overexpressed Flag-p27Xic1 and HA-aPKC-CAAX in *Xenopus* embryos and let them grow until neurula stage. The embryos were fixed, sectioned and immunostainied using anti-Flag and anti-HA antibody for detection of Flag-p27Xic1 and HA-aPKC-CAAX repectively. Immuno-staining showed that Flag-p27Xic1 (green) was largely in the nucleus of the cells, but a portion of aPKC-CAAX (red), which was mostly localized to the cell cortex, was also found in the nucleus (see the white arrows). These stainings led to the idea that aPKC interacts with p27Xic1 in the nucleus. White scale bar in A represents 50μM.

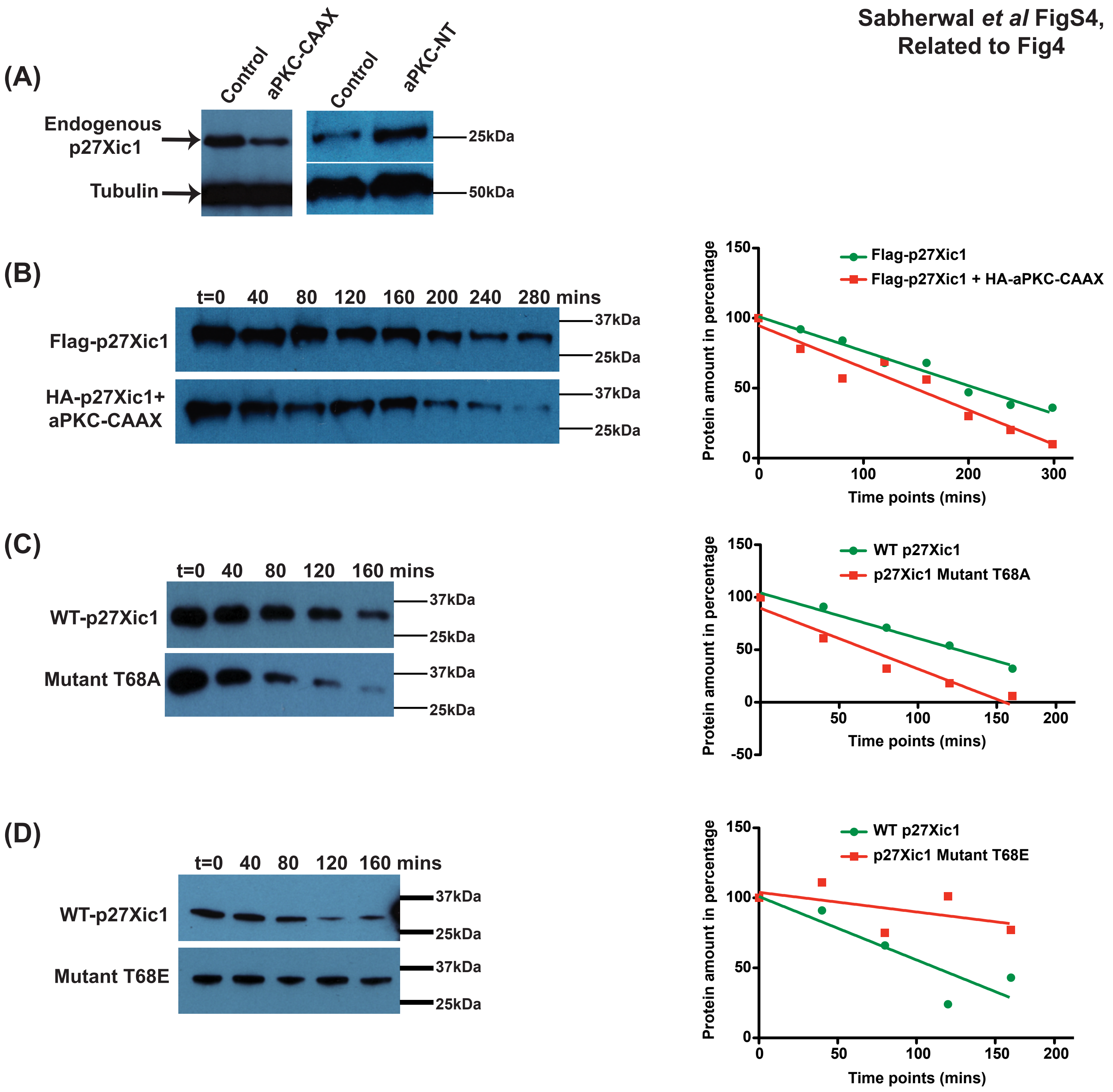

FigS4, related to fig4: Effects of aPKC on protein abundance and stability of p27Xic1. **(A)** Embryos over-expressing constitutively active aPKC (aPKC-CAAX) or dominant negative form of aPKC (aPKC-NT) show reduced or increased levels of endogenous p27Xic1, respectively. **(B)** Cycloheximide treatment of HeLa cells overexpressing Flag-p27Xic1 alone or in combination with HA-aPKC-CAAX shows that half-life of p27Xic1 is reduced in the presence of aPKC-CAAX, suggesting that p27Xic1 phosphorylation by aPKC might affect its half-life. **(C)** But contrary to the expectations, phospho-mutant of p27Xic1 (eg. T68A) showed protein destabilization, while protein for its phospho-mimetic mutant (T68E) showed enhanced stability **(D)**, leading to the idea that the effects of aPKC on the stability of p27Xic1 are complex and can not be mimicked by singlepoint mutations on p27Xic1.

Sabherwal *et al* FigS5
Related to Fig4C and D

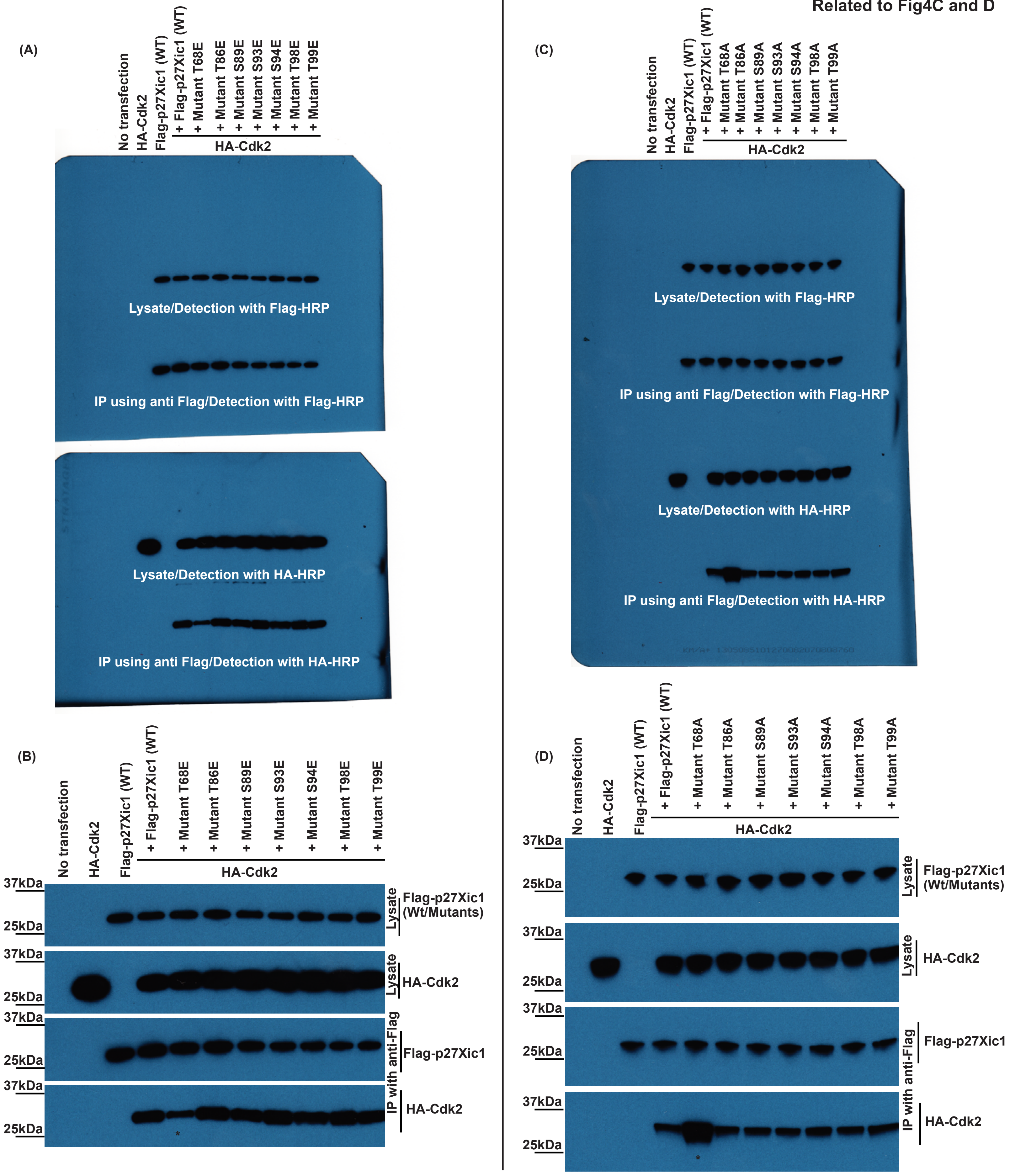

FigS5, related to fig4C and D: Blots on the left hand side show raw data (above) and processed data (below) for fig4C, while blots on the right hand side show raw data (above) and processed data (below) for fig4D.

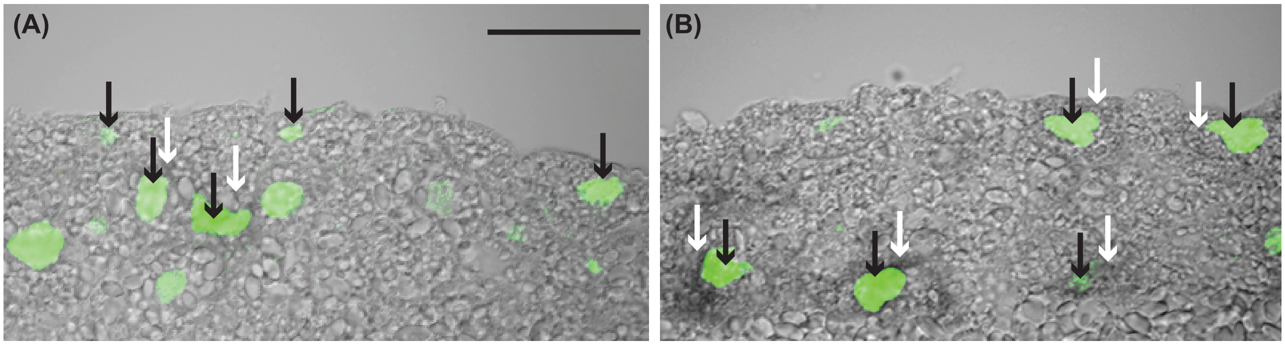

FigS6, related to fig5: (A) p27Xic1 promotes neurogenesis in a cell autonomous manner. To see if the effects of p27Xic1 on neurogenesis were cell autonomous or not, we combined the in situ hybridisation (non-fluorescent, black precipitation) against neuronal differentiation marker *N-tubulin* with immunostaining (fluorescent, green) against Flag-p27Xic1 using anti-Flag antibody, on embryos overexpressing Flag-p27Xic1. Sections from the embryos were imaged. As an example of the sections shows, overexpression of Flag-p27Xic1 (green, black arrow) promotes neurogenesis (*N-tubulin*, black precipitation, white arrow) within the same cells overexpressing p27xic1. In image 'A', outer superficial polarised cells show no *N-tubulin* expression even after Flag-p27Xic1 overexpression. **(B)** Phospho-mutant p27Xic1 can also promote neurogenesis in the outer superficial polarized cells as well. Cells overexpressing Phospho-mutant Flag-p27Xic1 (that can not be rescued by aPKC unlike wild type Flag-p27Xic1) promotes neurogenesis not only in the inner nonpolar cells but in the outer superficial polarised cells as well. The images also show that Flag-p27Xic1 protein is localised exclusively to the cell nucleus. Black scale bar in A represents 50μM.

Legends for supplemental movies

Movie S1A, related to fig2B – Time-lapse movie for HeLa Fucci biosensor treated with DMSO (control). Movie shows HeLa Fucci cells dividing in the presence of DMSO (control). Time-lapse images were analyzed and converted into AVI movies using ImageJ software. Appearance of an arrow marks the dividing cells in the movies. Cell cycle parameters were calculated as explained in supplemental experimental procedure 1. Please refer to fig2B for calculated parameters.

Movie S1B, related to fig2B – Time-lapse movie for HeLa Fucci biosensor treated with aPKC specific myristoylated pseudosubstrate inhibitor. Movie shows HeLa Fucci cells dividing in the presence of aPKC specific inhibitor (Myristoylated pseudosubstrate inhibitor/Myr PSI). Time-lapse images were analyzed and converted into AVI movies using ImageJ software. Appearance of an arrow marks the dividing cells in the movies. Cell cycle parameters were calculated as explained in supplemental experimental procedure 1. Please refer to fig2B for calculated parameters. The movie shows that on aPKC inhibition, Fucci cells exhibit longer cell cycle length (Tc) due to longer G1 phase length (T_{G1}) and longer S phase length (T_S).

Movie S1C, related to fig2B – Time-lapse movie for HeLa Fucci biosensor treated with aPKC inhibitor (GÖ6983/GO). Movie shows HeLa Fucci cells dividing in the presence of aPKC inhibitor (GÖ6983/GO). Time-lapse images were analyzed and converted into AVI movies using ImageJ software. Appearance of an arrow marks the dividing cells in the movies. Cell cycle parameters were calculated as explained in supplemental experimental procedure 1. Please refer to fig2B for

calculated parameters. The movie shows that on aPKC inhibition, Fucci cells exhibit longer cell cycle length (Tc) due to longer G1 phase length (T_{G1}) and longer S phase length (T_S).

Supplemental experimental procedure

1. Estimation of cell cycle kinetics parameters using DPSL, PLM and Fucci biosensor analysis DPSL was applied to embryos to estimate total cell cycle length (T_C) and S-phase length (T_S), for polar and nonpolar Sox3+ progenitors and for injected vs noninjected sides (Fig1Ac, 2Ac). PLM performed for the duration of 240 mins gave us an estimate of the length of G2+1/2M-phases (Fig1Bc). Mitotic index information in combination with T_C estimated by DPSL was used to estimate T_M (time for mitosis) for both progenitors (Fig1Bd). T_M for polar progenitors calculated this way matched with the T_M calculated by live imaging (data not shown). Estimated T_M values in combination with the length of G2+1/2M gave estimation for the length of G2. Finally, length of G1 phase was calculated by deducting lengths of S, G2 and M phases from the length of the total cell cycle [$T_{G1}=T_C-(T_S+T_M+T_{G2})$].

For the purpose of estimating cell cycle kinetics parameters on cells, HeLa Fucci live cells were imaged as described before (Sakaue-Sawano et al., 2008). Fucci cells can be used visually to calculate the exact length of different cell cycle phases (G1 with Red fluorescence, early S with Yellow fluorescence, SG2M with Green fluorescence and M with round shaped cells, schematic in Fig2B) in control and experimental condition. Roughly 120cells/well were plated on a 96-well glass-bottom dish on day0. On day1, normal DMEM media was replaced with movie media (Nutrient Mixture F-12 Ham, Sigma Aldrich) containing appropriate amount of chemical inhibitor. On day2, control and experimental cells were imaged on a heated stage at 37^0C and 5% CO_2 supply using NikonA1 confocal microscope for 2-3 days. Images were captured every 20 mins. Collected images were converted into time-lapse movies and analyzed using ImageJ software, for estimating the lengths of various cell cycle phases.

2. Plasmid constructs Constructs relating to aPKC are already described (Sabherwal et al., 2009). For the p27Xic1 constructs (pGEX-6P-GST-p27Xic1, pCS2-3Flag-p27Xic1 and deletion constructs used in immuno-complex kinase assays), desired coding regions were PCR-amplified using clone TTpA019B19 from neurula stage *Xenopus tropicalis* EST library as template and cloned into appropriate vectors. Phospho- and phospho-mimetic mutants against the sites identified by mass-spectrometry analysis of p27Xic1 protein post *in vitro*- and *in vivo*-kinase assays were generated using Quick Change site-directed mutagenesis kit (Agilent Technologies), following manufacturer's guidelines. Full-length coding regions for Cdk2 and Cdk4 were PCR-amplified using clones TNeu043b09 and TNeu018k23, respectively, from neurula stage *X. tropicalis* library and cloned into pCS2-HA expression vector.

3. Protein half-life assay HeLa cells overexpressing p27Xic1 alone or in combination with aPKC-CAAX were treated with 100µM Cycloheximide (Sigma Aldrich) for the designated time periods and protein half-life assays were performed as described before (Scott, Ingram, & Ball, 2002). Please refer to figS3 for data.

4. Sample preparation for mass-spectrometry analysis To prepare samples for MS analysis, HeLa cells grown to 80-90% confluency in 10cm cell culture dishes were transfected with Flag-p27Xic1 with HA-aPKC-CAAX (for *in vivo* kinase sample), or alone (for control for *in vivo* kinase sample, or for *in vitro* kinase reaction). 24hrs post transfection, cells were harvested and lysed in 1ml of lysis buffer containing protease and phosphatase inhibitors (Thermo Scientific). Flag-p27Xic1 was immunoprecipitated (IPed) as described in materials and methods, using 25µl of ProteinA/G beads and 100µg of anti-Flag antibody. For *in vitro* kinase reaction, IPed Flag-p27Xic1 was mixed with 1µg of His-aPKC (Millipore) and cold ATP as

described in materials and methods. IPed Flag-p27Xic1 was run on 10% gel. Post run, gel was stained using Coomassie based InstantBlue solution (Expedeon) following manufacturer's instructions.
